# Aglycemic growth enhances carbohydrate metabolism and induces sensitivity to menadione in cultured tumor-derived cells

**DOI:** 10.1186/s40170-021-00241-0

**Published:** 2021-01-19

**Authors:** Cameron A. Schmidt, Kelsey L. McLaughlin, Ilya N. Boykov, Rafiq Mojalagbe, Arthi Ranganathan, Katherine A. Buddo, Chien-Te Lin, Kelsey H. Fisher-Wellman, P. Darrell Neufer

**Affiliations:** 1 East Carolina Diabetes and Obesity Institute, Greenville, NC USA; 2 Dept. of Physiology, Brody School of Medicine, East Carolina University, Greenville, NC USA

**Keywords:** Cancer, Mitochondria, Oxidative phosphorylation, Glycolysis, Confocal microscopy, Oroboros, Seahorse xf24, Galactose, HEPG2

## Abstract

**Background:**

Hepatocellular carcinoma (HCC) is the most prevalent form of liver malignancy and carries poor prognoses due to late presentation of symptoms. Treatment of late-stage HCC relies heavily on chemotherapeutics, many of which target cellular energy metabolism. A key platform for testing candidate chemotherapeutic compounds is the intrahepatic orthotopic xenograft (IOX) model in rodents. Translational efficacy from the IOX model to clinical use is limited (in part) by variation in the metabolic phenotypes of the tumor-derived cells that can be induced by selective adaptation to subculture conditions.

**Methods:**

In this study, a detailed multilevel systems approach combining microscopy, respirometry, potentiometry, and extracellular flux analysis (EFA) was utilized to examine metabolic adaptations that occur under aglycemic growth media conditions in HCC-derived (HEPG2) cells. We hypothesized that aglycemic growth would result in adaptive “aerobic poise” characterized by enhanced capacity for oxidative phosphorylation over a range of physiological energetic demand states.

**Results:**

Aglycemic growth did not invoke adaptive changes in mitochondrial content, network complexity, or intrinsic functional capacity/efficiency. In intact cells, aglycemic growth markedly enhanced fermentative glycolytic substrate-level phosphorylation during glucose refeeding and enhanced responsiveness of both fermentation and oxidative phosphorylation to stimulated energy demand. Additionally, aglycemic growth induced sensitivity of HEPG2 cells to the provitamin menadione at a 25-fold lower dose compared to control cells.

**Conclusions:**

These findings indicate that growth media conditions have substantial effects on the energy metabolism of subcultured tumor-derived cells, which may have significant implications for chemotherapeutic sensitivity during incorporation in IOX testing panels. Additionally, the metabolic phenotyping approach used in this study provides a practical workflow that can be incorporated with IOX screening practices to aid in deciphering the metabolic underpinnings of chemotherapeutic drug sensitivity.

**Supplementary Information:**

The online version contains supplementary material available at 10.1186/s40170-021-00241-0.

## Background

Hepatocellular carcinoma (HCC) is the most common form of liver malignancy, comprising the majority of primary liver cancer incidence worldwide [1]. Because HCC is a comorbidity of fatty liver disease and diabetes, incidence is expected to rise as the population of patients with metabolic disease grows globally [1]. Current treatment methods for HCC rely heavily on liver resection and chemotherapeutic modalities [1]. However, the efficacy of these treatments depends on early detection of the disease, which often does not become symptomatic until late stages [2].

Though chemotherapeutic drugs often have multiple associated mechanisms of action, metabolic liabilities are commonly suggested to be important for their anti-cancer effects. For example, sorafenib, doxorubicin, and etoposide have all been shown to alter mitochondrial respiratory function in cancer cells [3–5]. Additionally, many new drugs (e.g., sorafenib derivatives), which are also likely to have metabolic implications, are being developed for use in late-stage HCC and other cancers [1, 2, 6, 7]. A common platform for testing chemotherapeutic strategies for HCC treatment is the rodent intrahepatic orthotopic xenograft (IOX) model [8–10]. Unfortunately, promising candidate drugs identified in rodent models often fail in follow-up clinical trials [8]. Poor translation from preclinical testing to clinical efficacy is a complex phenomenon with many confounding variables. However, one potentially important factor is variation in the underlying metabolic phenotypes of the cultured tumor-derived cells that are used in IOX testing panels [8].

Tumor-derived cell lines have variable degrees of metabolic flexibility when challenged with toxicants (e.g., 2-deoxy-glucose, 3-bromo-pyruvic acid, dinitrophenol, metformin) [11–14]. To compensate for these effects, IOX studies often use several different cell lines, which has resulted in reports of variable drug efficacy that depends (in part) on the metabolic phenotype of the source cell line [8]. Though tumor-derived cell metabolic phenotype can be influenced by many biological variables, one particularly important factor is selective adaptation to growth conditions during subculture [15, 16], the effects of which are readily mediated by a multitude of commercially available media formulations and supplements [16]. Selective growth conditions have been used to induce metabolic sensitivities [6, 17, 18] and screen for inborn errors of metabolism [15]. However, metabolic adaptations that arise in response to growth conditions are rarely examined explicitly, thus limiting the quality of the IOX model and complicating the interpretation of studies to identify metabolic toxicants.

In this study, a detailed multilevel systems approach was used to define specific bioenergetic adaptations to aglycemic growth conditions in HEPG2 cells. The HEPG2 tumor-derived cell line was selected because it is commonly included in IOX testing panels and is supported by an extensive body of comparative literature [13, 19–28]. The approach encompasses parallel assessment at the organelle and whole-cell levels, mitochondrial structural and functional measurements, selective substrate refeeding to target specific modes of central carbon metabolism, and comparison of metabolic fluxes under different states of energetic demand. Based on the observations of previous studies [6, 17, 18], it was hypothesized that aglycemic growth conditions would facilitate compensatory enhancement of oxidative metabolism and repression of aerobic fermentative metabolism compared to HEPG2 cells subcultured in glycemic growth conditions.

## Methods

### Materials and reagents

All chemicals and reagents were obtained from Sigma-Aldrich (St Louis, MO, USA). Several specific culture media were used in this study: High-glucose DMEM (DMEM-H-GLC) consisted of phenol-free Dulbecco’s modified Eagle medium (DMEM) supplemented with 25 mM d-glucose, 4 mM l-glutamine, 1 mM pyruvate, 10% fetal bovine serum (Gibco), and penicillin/streptomycin (Gibco; 50 U/mL and 50 μg/mL respectively). High-galactose DMEM (DMEM-H-GAL) was the same formulation as DMEM-GLC, with 25 mM galactose in place of glucose. Low-glucose DMEM (DMEM-L-GLC) consisted of phenol-free Dulbecco’s modified Eagle medium (DMEM) supplemented with 5 mM d-glucose, 1 mM l-glutamine, 250 μM pyruvate, 10% fetal bovine serum (Gibco), penicillin/streptomycin (Gibco; 50 U/mL and 50 μg/mL respectively). Unbuffered DMEM-H-GLC or DMEM-H-GAL were the same formulation described for each, but without any pH buffering components. Unbuffered substrate-free DMEM (DMEM-U-SF) consisted of phenol-free DMEM supplemented with 10% fetal bovine serum (Gibco), penicillin/streptomycin (Gibco; 50 U/mL and 50 μg/mL, respectively) (no glucose/galactose, pyruvate, or glutamine was included). Buffered substrate-free DMEM (DMEM-B-SF) was the same as the unbuffered media, but with 20 mM HEPES. All media was adjusted to pH 7.4 at 37 °C.

### HEPG2 cell subculture

Human hepatocellular carcinoma cells (HEPG2) were obtained from ATCC (Manassas, VA, USA). Cells were cultured for three passages (three population doubling periods) in DMEM-H-GLC. To induce adaptation to aglycemic conditions, cells were switched to DMEM-H-GAL and grown for an additional three passages. Control cells were grown in DMEM-H-GLC concomitantly. The two growth condition-adapted cell lines are denoted: HEPG2-Glc (glucose) or HEPG2-Gal (galactose). Cells were then frozen in working stocks and stored in a liquid nitrogen dewar until thawed for experiments. All experiments were performed using cells at passage six from those initially obtained from ATCC.

### Laser scanning confocal microscopy

Cells were seeded at 8 × 10^5^ cells/well in #1.5 glass-bottom 6-well culture dishes (MatTek, Ashland, MA, USA) and were grown for 48 h in DMEM-GLC or DMEM-GAL as appropriate. One hour prior to imaging, cells were washed and incubated with DMEM-GLC or DMEM-GAL, containing 2 μM Hoechst and 250 nM mitotracker green-FM (Molecular Probes, Eugene, OR). Cells were then rinsed in 1X PBS and changed to DMEM-B-SF containing 50 nM tetramethyl rhodamine methyl ester (TMRM) and were incubated in a CO_2_-free incubator for 1 h. All imaging were performed using an Olympus FV1000 laser scanning confocal microscope (LSCM) with an onstage incubator at 37 °C. Acquisition software was Olympus FluoView FSW (V4.2). The objective used was × 60 oil immersion (NA = 1.35, Olympus Plan Apochromat UPLSAPO60X(F)). Images were 800 × 800 pixel with 2 μs/pixel dwell time, sequential scan mode, with a × 2.5 digital zoom. Mitotracker green-FM was excited using the 488-nm line of a multiline argon laser; emission was filtered using a 560-nm dichroic mirror and 505–540-nm barrier filter. TMRM was excited using a 559-nm laser diode; emission was filtered using a 575–675-nm barrier filter. Zero detector offset was used for all images, and gain at the detectors was kept the same for all imaging. The pinhole aperture diameter was set to 105 μm (1 Airy disc).

### Mitochondrial volumetric analysis

TMRM channel images were selected for volumetric analysis because of the dye’s higher signal to background ratio (compared with MitoTracker Green-FM) and were analyzed using ImageJ [29]. Sixteen bit images were made into a composite. Circular ROIs were manually selected using the ROI manager plugin. Images were then decomposed into separate 16-bit image stacks leaving the ROI positions intact. A Huang auto threshold was used for automated selection of signal for all three channels. Following threshold application, each signal was measured using the multi-measure feature. Only whole cells were analyzed (i.e., cells on edges of the FOV were excluded).

The following calculations were performed to determine the relevant signal volumes.

1 $$ \mathrm{Signal}\ \mathrm{volume}\ \left({\upmu \mathrm{m}}^3\right)=\frac{\sum A\bullet Z}{N} $$

where *A* is the signal positive area selected using a Huang auto threshold (μm^2^), *Z* is the optical section thickness (axial resolution; μm), and *N* is the number of steps within each optical section (i.e., axial resolution divided by the step size). The latter operation is necessary to correct for oversampling of the signal volumes.

### Mitochondrial network complexity

Analysis was performed on images from the TMRM channel, using a method that was closely adapted from two previous studies [30, 31]. This method was shown to be robust to variation in thresholding values during segmentation and was also sensitive to experimentally induced changes in mitochondrial network architecture [30, 31]. TMRM channel images were selected for network complexity analysis because of the dye’s higher signal to background ratio (compared with MitoTracker Green-FM). Briefly, spatial resolution was determined (and point spread function measured) using sub-resolution fluorescent beads (PS Speck; ThermoFisher, Waltham, MA, USA). Curve fitting was performed using the MetroloJ plugin in ImageJ. Image stacks were trimmed to an ~ 2.5-μm-thick z-stack range from the middle of the cells. Image deconvolution was performed using the Richardson-Lucy algorithm (*N* = 100 iterations) in the DeconvolutionLab2 plugin in ImageJ [32]. Summed intensity Z-projections were made from the deconvolved image stacks, and a custom tophat spatial filter [31] was applied to enhance the pixel intensities between background and signal. Network complexity parameters were determined using Matlab (Version R2020a). Images were binarized (im2bw routine; standard threshold value = 0.75) and skeletonized (bwmorph routine), and the connectivity of clusters was determined using a nearest neighbor search routine (bwconncomp; *k* = 8); clusters less than three pixels in size were removed to reduce noise (bwareaopen), and cluster mass in pixels was determined for all clusters in each cell (regionprops routine). Probability of observing a cluster of mass (*M*) was determined from the relative frequency distributions of cluster masses in each cell analyzed. Cluster entropy is defined as previously described [30]:

2 $$ H=\sum {P}_i(M)\mathrm{Log}\left({P}_i(M)\right) $$

where *P*_*i*_*(M)* is the *i* th relative frequency of a cluster of mass *M* in pixels. RandClust and SingleClust entropy values were determined by simulating a cell in which an equal frequency of cluster masses was observed among all possible values, or > 99% of cluster masses fell within a single value (respectively).

### Electrical potential across the inner mitochondrial membrane (ΔΨm) in live intact cells

ΔΨ_m_ was determined using non-quenching concentrations of tetramethylrhodamine methyl ester (TMRM) [33–35]. In non-quenching mode, low concentrations of TMRM equilibrate directly with variation in ΔΨ_m_, as opposed to quench mode in which accumulating TMRM forms aggregates that reduce signal intensity, which is positively correlated with ΔΨ_m_ [33]. As an additional control, carbonyl cyanide 4-(trifluoromethoxy)phenylhydrazone (FCCP; 1 μM) was used to fully depolarize the membrane potential. Because TMRM is used in the non-quenched mode, FCCP addition leads to a decrease in fluorescence such that it is similar to background (SFigure 5). Images were analyzed using ImageJ [29]. Out-of-field images from the axial dimension were trimmed from each stack. Image stacks were background-corrected using darkfield images obtained each day of imaging. Median background intensity (*I*_B_) was measured by inverting a Huang auto threshold for each image stack. The estimated electrical potential difference relative to the median value of the extra-mitochondrial signal was then mapped onto each pixel in each image using a math macro:

3 $$ \Delta  {\Psi}_m\ \left(\mathrm{mV}\right)=2.303\ast \frac{\mathrm{RT}}{zF}\ast \log \left(\frac{\mathrm{v}}{I_B}\right) $$

where *R* is the universal gas constant (J/mol*K), *T* is the absolute temperature (310.15K), *z* is the + 1 charge of TMRM, F is Faraday’s constant (C), and *v* is the gray value of the designated pixel. A Huang auto threshold was then applied to each image in the stack, and median and maximum values for each threshold were obtained. This measurement method reduced biasing of the total measured signal toward low-intensity pixel values. The median and max values for the cells in each stack were averaged for data representation.

### Mitochondrial isolation

Differential centrifugation was employed to prepare isolated mitochondria from cultured cells. The buffers used for all isolations were as follows: buffer A–MOPS (50 mM; pH = 7.1), KCl (100 mM), EGTA (1 mM), MgSO4 (5 mM); buffer B–buffer A, supplemented with bovine serum albumin (BSA; 2 g/L). Cells were trypsinized at 37 °C for ~ 5 min, then growth media was added to stop the trypsin reaction, and the cells were centrifuged down at 300×*g* at room temperature. Growth media was then aspirated, and the pellet was resuspended in phosphate-buffered saline (PBS) and spun again at 300×*g*. The pellet was then resuspended in ice-cold buffer B, and the cells were homogenized via a Teflon pestle and borosilicate glass vessel for 40 passes. The homogenate was centrifuged at 800×*g* for 10 min at 4 °C. The supernatant was then pipetted into a separate tube, and the pellet was again resuspended in buffer B, homogenized, and centrifuged at 800×*g*. This process was repeated a total of 3 times, and the supernatants from each of the 3 rounds of homogenization were pooled together and centrifuged at 10,000×*g* for 10 min at 4 °C. The mitochondrial pellet was then washed in buffer A, transferred to a microcentrifuge tube, and centrifuged again at 10,000×*g* for 10 min at 4 °C. Buffer A was aspirated from each tube, and final mitochondrial pellets were suspended in 100–150 μL of buffer A. Protein content was determined via the Pierce BCA protein assay.

### Mitochondrial functional assessment

Functional assays involving isolated mitochondria were carried out in buffer C: potassium-MES (105 mM; pH = 7.2), KCl (30 mM), KH_2_PO_4_ (10 mM), MgCl2 (5 mM), EGTA (1 mM), BSA (2.5 g/L), and creatine (Cr; 5 mM). High-resolution O_2_ consumption measurements were conducted using the Oroboros Oxygraph-2K (O2K; Oroboros Instruments). All respiration experiments were carried out at 37 °C in a 1 mL reaction volume. Fluorometry experiments were carried out using a QuantaMaster Spectrofluorometer (QM-400; Horiba Scientific) at 37 °C in a 0.2 mL reaction volume with continuous stirring. The following substrate conditions were tested for all functional assays except for the substrate preference assay: pyruvate/malate (Pyr/M; 5/2 mM) and glutamate/malate (G/M; 10/2 mM). Several important quality assessment measures were performed that should be emphasized: (1) damage to the mitochondrial outer membrane was assessed by determining the cytochrome c flux control efficiency [36]. Ten micromolar cytochrome c was included in the assays to ensure that cytochrome c was not limiting to respiration. (2) Citrate synthase activity was assessed to ensure that the preparations contained similarly pure mitochondrial fractions (described in more detail in the “Citrate synthase activity” section). (3) Respiratory control ratios [36], as well as sensitivity to controlled changes in adenylate concentration, were determined using the creatine kinase free energy clamp (described in more detail in the “Force flow” section).

### Citrate synthase activity

Citrate synthase (CS) activity was determined using a colorimetric plate-based assay in which CoA-SH, a byproduct formed by the CS-mediated reaction of oxaloacetate and acetyl-CoA, interacts with 5′, 5′-dithiobis 2-nitrobenzoic acid (DTNB) to form TNB (OD: 412 nm). Assay buffer consisted of buffer C (105 mM potassium-MES, 30 mM KCl, 10 mM KH2PO4, 5 mM MgCl2, and 1 mM EGTA; pH = 7.2) supplemented with DTNB (0.2 mM) and acetyl-CoA (0.5 mM). A 96-well round bottom plate was loaded with assay buffer (200 μL/well), the permeabilizing agent alamethicin (0.03 mg/mL), and isolated mitochondria (10 μg/well) were added and then incubated at 37 °C for 5 min to deplete endogenous substrates. The assay was initiated by the addition of oxaloacetate (1 mM) to sample wells, with absorbance at 412 nm recorded every 30 s for 20 min. The mitochondrial suspension was also added to one control well per sample to account for nonspecific activity, which was later subtracted from the sample rate. CS activity was determined using the Beer-Lambert Law and the molar absorption coefficient of TNB (13.6 mM/cm).

### Substrate preference assay

Differences in substrate preference between the two feeding conditions were assessed using the steady-state O_2_ consumption rates (*J*O_2_) following sequential additions of different carbon substrates and specific inhibitors, in the presence of a saturating steady-state ADP concentration of 300 μM. This experiment was conducted in buffer C supplemented with cytochrome C (CytC; 10 μM), HK (1 U/mL), and glucose (5 mM). Mitochondria (0.05 mg/ml) were added to the buffer, followed by ADP and pyruvate/malate (1/2 mM). The pyruvate carrier inhibitor UK5099 was then added to prevent pyruvate oxidation, followed by the addition of glutamate (10 mM). NADH-linked respiration was then inhibited using rotenone (0.05 μM), and respiration was stimulated using glycerol-3-phosphate (G3P; 10 mM). Succinate was then added to stimulate complex II-linked respiration (10 mM). Complex III was then inhibited by the addition of antimycin A (0.005 μM), and respiratory flux through complex IV was stimulated through the addition of the electron donor TMPD dissolved in 2 M ascorbate (0.5 μM).

### Force flow

Steady-state *J*O_2_ was determined within individual force-flow experiments using a modified version of the creatine kinase energetic clamp technique [37–39]. This assay is based upon the calculation of the free energy of adenosine triphosphate (ATP) hydrolysis (ΔG′_ATP_, written throughout the manuscript as ΔG_ATP_) from known amounts of Cr, phosphocreatine (PCr), and ATP in the presence of excess amounts of creatine kinase (CK), using the equilibrium constant for the CK reaction (i.e., *K*_CK_). ΔG_ATP_ was calculated according to the following formula:

4 $$ \Delta G{\prime}_{\mathrm{ATP}}=\Delta G{\prime}_{\mathrm{ATP}}^{\circ }+ RT\ln \frac{\left[\mathrm{Cr}\right]\left[{\mathrm{P}}_i\right]}{\left[\mathrm{PCr}\right]\left[K{\prime}_{\mathrm{CK}}\right]} $$

where ΔG′°_ATP_ is the standard apparent transformed Gibbs energy (under a specified pH, ionic strength, free magnesium, and pressure), *R* is the gas constant (8.3145 J/kmol), and *T* is temperature in Kelvin (310.15). For complete details regarding the calculation of ΔG′_ATP_ at each titration point, see [40]. To begin, isolated mitochondria (0.05 mg/ml) were added to buffer C supplemented with CytC (10 μM), followed by the addition of respiratory substrates to bring respiration to state 4. The CK clamp was then initiated by the addition of ATP (5 mM), PCr (1 mM), and CK (20 U/mL). Sequential additions of PCr to 6, 15, and 21 mM were then performed to gradually slow *J*O_2_ back toward baseline. After the final PCr addition, the uncoupler FCCP was then titrated (0.5, 1, 2 μM) to stimulate respiration back up toward maximal *J*O_2_. Plotting the calculated ΔG_ATP_ against the corresponding *J*O_2_ depicts a force-flow relationship, the slope of which represents the conductance/sensitivity of the entire respiratory system under specified substrate constraints as previously described [37, 38, 40].

### Mitochondrial membrane potential (ΔΨ) and NAD(P)H/NAD(P)+ redox

Fluorescent determination of ΔΨ and NAD(P)H/NAD(P)^+^ throughout the force-flow experiment was carried out in parallel using the QM-400. Determination of ΔΨ via TMRM was done by recording the fluorescence ratio of the following excitation/emission parameters: Ex/Em, (576/590)/(552/590) [34]. A KCl standard curve was then used to convert the 576/552 ratio to millivolts. The KCl standard curve was performed in the presence of valinomycin as described previously [40]. In this protocol, isolated mitochondria energized with succinate/rotenone (Succ/R; 10 mM/0.05 μM) were incubated in a potassium-free buffer in the presence of valinomycin, a potassium-specific ionophore. ΔΨ can be reasonably estimated by applying the Nernst equation and buffer ion concentrations resulting from sequential additions of KCl, assuming a starting matrix potassium concentration of 120 mM [40]. NAD(P)H excitation/emission parameters were 350/450. Buffer for all assays was buffer C, supplemented with CytC (10 μM) and TMRM (0.2 μM). To begin, isolated mitochondria (0.05mg/ml) were added to the assay buffer, followed by the addition of respiratory substrates, CK clamp components, and then sequential PCr additions to 6, 15, and 21 mM as in the respiratory experiment. Following the final PCr addition, cyanide (10 mM) was added to induce a state of 100% reduction within the NAD(P)H/NAD(P)^+^ couple. The fluorescence (Ex/Em, 350/450) signal recorded in the presence of mitochondria alone without respiratory substrates was used as the 0% reduction state for the NAD(P)H/NAD(P)^+^ couple. NAD(P)H/NAD(P)^+^ during the entire experiment was expressed as a percentage of reduction according to the following formula:

5 $$ \%\mathrm{Reduction}=\frac{F-{F}_{0\%}}{F_{100\%}-{F}_{0\%}} $$

### Measurement of ATP production rates (*J*ATP) and ATP/O ratios

Parallel respiration and fluorometric experiments were carried out in order to generate an ATP/O ratio. Both sets of experiments were conducted in buffer C supplemented with CytC (10 μM), Ap5A (0.15 μM), hexokinase (HK; 1 U/mL), glucose (5 mM), glucose-6-phosphate dehydrogenase (2 U/mL), and NADP^+^ (4 mM). The rate of change in NAD(P)H fluorescence (Ex/Em, 350/450) in the QM-400 experiments was equated to the rate of ATP production by the mitochondria (*J*ATP), as has been previously described [41]. Experiments began with the addition of mitochondria (0.05 mg/ml for both) to the modified buffer C, followed by the addition of respiratory substrates. ADP was then titrated in to 10 and 500 μM. Fluorometry experiments were then ended by the addition of oligomycin (oligo; 0.02 μM). Respiration experiments continued with an FCCP titration (0.5, 1, 2 μM) following the final ADP addition. The ATP/O ratio was then calculated using the ratio of the *J*ATP and the steady-state *J*O_2_ at each addition point, divided by 2.

### Live cell extracellular flux analysis with substrate refeeding

Extracellular oxygen flux (*J*O_2_) and acidification rate (ECAR) were measured using a Seahorse XF24e flux analyzer (Agilent Technologies, Santa Clara, CA, USA). Cells were seeded at 2 × 10^5 cells/well, 48 h prior to running assays. Seeded cells were rinsed with 1X PBS and media was changed to DMEM-U-SF (pH 7.4 at 37 °C). Cells were then incubated for one hour in a CO_2_-free incubator. The flux analysis protocol was as follows: basal OCR and ECAR were measured in unbuffered DMEM, followed by carbon fuel substrate refeeding (25 mM glucose, 4 mM glutamine, or 1 mM pyruvate at standardized volumes with a 3 min mixing step prior to measurement). Five micromolar oligomycin A was then injected to inhibit ATP-coupled respiration, FCCP (1 μM) was injected to increase H^+^ conductance across the inner mitochondrial membrane, and rotenone and antimycin A (5 μM) were injected to inhibit electron transport system flux at complexes I and III, respectively. Immediately following the experimental protocol, plates were removed, and cells were lysed in radioimmunoprecipitation (RIPA) buffer at 4 °C. Protein concentration in each well was determined using a bicinchoninic acid (BCA) protein assay (Thermo Fisher).

### ECAR unit conversion and *J*ATP_Glyc_ rate estimation

ECAR measurements are often interpreted as direct measurements of lactic acid production derived from reduction of pyruvate [42]. However, these units are difficult to interpret in the context of cellular metabolism because they are not stoichiometrically linked with lactic acid production rates. Here, the method described by Mookerjee et al. was used to convert the ECAR (pH) measurements (mpH ∙ min^−1^ ∙ μg protein^−1^) to a proton efflux rate (*J*H^+^) [43]. The buffering capacity (C; mpH ∙ pmol^−1^ H^+^) of the DMEM-U-SF medium was measured at 37°C in bulk solution by titrating known amounts of hydrochloric acid (HCl) and was determined to be .094 mpH ∙ pmol^−1^ H^+^. The unit conversion was performed as follows:

6 $$ J{\mathrm{H}}^{+}=\frac{\mathrm{ECAR}}{C} $$

To estimate the ATP production rate (*J*ATP_Glyc_) due to glycolytic flux, the *J*H^+^ described in Eq. (6) was multiplied by the stoichiometric coefficient 1 pmol ATP/pmol lactic acid-derived H^+^.

### Oxygen consumption rate correction and *J*ATP_OxPhos_ rate estimation

Mitochondrial-specific oxygen consumption rate (*J*O_2,Mito_) was determined by:

7 $$ J{O}_{2,\mathrm{Mito}}=J{O}_{2,M}-J{O}_{2\mathrm{Rot}/\mathrm{AmA}} $$

where *J*O_2M_ is the measured *J*O_2_ and *J*O_2Rot/AmA_ is in the presence of 5 μM rotenone and 5 μM antimycin A. This corrects for non-mitochondrial OCR.

Apparent coupling efficiency (*Q*) was determined using the fractional change between the basal mitochondria-specific *J*O_2_ and the *J*O_2_ in the presence of the F_o_F_1_ ATPase inhibitor oligomycin A (5 μM).

8 $$ Q=\left({JO}_{2,\mathrm{Mito}}-{JO}_{2,\mathrm{Oligo}}\right)/{JO}_{2,\mathrm{Mito}} $$

To estimate ATP production rates due to mitochondrial respiration (*J*ATP_OxPhos_), *J*O_2_ values were multiplied by the measured coupling efficiency, and the estimated ATP/O ratios provided in Table 1, similar to a previously described method [44].

9 $$ J{\mathrm{ATP}}_{\mathrm{O}\mathrm{xPhos}}=J{{\mathrm{O}}_2}_{\mathrm{Mito}}\bullet Q\bullet 2\frac{\mathrm{mole}\ \mathrm{O}}{\mathrm{mole}\ {\mathrm{O}}_2}\bullet \frac{\mathrm{ATP}}{{\mathrm{O}}_{\mathrm{O}\mathrm{xPhos}}}\ \left(\mathrm{Substrate}\ \mathrm{Specific}\right) $$

**Table 1:** ATP/O values measured in isolated mitochondria

Source cells	Substrates (mM)	Clamped (ADP) (μM)	Mean ATP/O ratio (SD)
HEPG2-Glc	Pyruvate/malate	10	0.95 (0.47)
	Glutamate/malate	10	0.98 (0.44)
	Pyruvate/malate	500	2.41 (1.2)
	Glutamate/malate	500	1.64 (0.47)
HEPG2-Gal	Pyruvate/malate	10	1.16 (.34)
	Glutamate/malate	10	1.01 (0.48)
	Pyruvate/malate	500	1.76 (.32)
	Glutamate/malate	500	1.68 (0.47)

Ketogenesis was assumed not to play a large role in HEPG2 cell carbon flux due to reports of low expression levels of ketogenic enzymes in HEPG2 cells as well as limited amounts of ketogenic amino acids and absence of free fatty acids in the media formulation [45, 46].

### Stimulation of acute metabolic demand

Monensin is a polyether antibiotic and ionophore that can be used to partially depolarize the plasma membrane sodium potential resulting in an energy-dissipating cycle between freely diffusing ionophore and the Na^+^/K^+^ ATPase [47, 48]. Extracellular oxygen flux (*J*O_2_) and acidification rate (ECAR) were measured as described above. The flux analysis protocol was as follows: basal OCR and ECAR were measured in unbuffered DMEM, followed by carbon fuel substrate refeeding (25 mM glucose, 4 mM glutamine, or 1 mM pyruvate at standardized volumes), 20 μM monensin A was then injected to cause an acute energetic demand, 0.1 mM ouabain was injected to inhibit the Na^+^/K^+^ ATPase, and rotenone and antimycin A (5 μM) were injected to inhibit electron transport system flux at complexes I and III, respectively. Immediately following the experimental protocol, plates were removed, and cells were lysed in radioimmunoprecipitation (RIPA) buffer at 4 °C. Protein concentration in each well was determined using a bicinchoninic acid (BCA) protein assay (Thermo Fisher). *J*H^+^ and ATP production rates were determined as described above. Specific APRs were calculated as a change from the monensin-induced rate, corrected for the ouabain rate:

10 $$ S\mathrm{timulated}\ J\mathrm{ATP}=\left({\mathrm{JATP}}_{\mathrm{Monensin}}-{J\mathrm{ATP}}_{\mathrm{Substrate}}\right)-\left({J\mathrm{ATP}}_{\mathrm{Ouabain}}-{J\mathrm{ATP}}_{\mathrm{Substrate}}\right) $$

### Cytotoxicity assays

Cells were seeded at 1.5 × 10^5^ cells/well in plastic 96-well culture dishes (MatTek, Ashland, MA, USA), and were grown for 48 h in DMEM-GLC or DMEM-GAL as appropriate. Cells were switched to DMEM-L-GLC containing 5-fold serial dilutions of the following compounds: 2-deoxy-glucose, dimethyl biguanide (metformin), oligomycin, or menadione. Following a 24-h incubation period, cells were rinsed and switched to compound-free DMEM-L-GLC. Cell viability was determined by quantitating the rate of reduction of resazurin dye to its fluorescent product resorufin, which is proportional to diaphorase activity in live cells [49]. The absolute rate of dye reduction was determined by calibrating to a standard curve derived by reacting known concentrations of dye (0–80 μM) with saturating (4 mM) ascorbate. The 560/590 nm fluorescence was measured using a microtiter plate reader (Biotek, Winooski, VT). Fluorescence was measured over a 2-h period at 37 °C. Dye reduction rates were linear for all samples. All chemical compounds and relevant metabolites were tested with the dye to ensure that false positive dye reduction would not occur under the measured conditions. Assays were repeated in duplicate on three separate occasions for each treatment/group (*N* = 6).

### Statistics

Data were analyzed using GraphPad Prism (Version 8.4.2). Data are represented by mean ± SEM. For univariate designs, means were compared using a two-tailed Student’s *t* test. For multivariate designs, means were compared using two-way ANOVA with Sidak’s multiple comparison test. Assumption of equal variance was tested using a Brown-Forsythe test. *P* < 0.05 were considered statistically significant.

## Results

### Aglycemic growth adaptation did not result in changes in mitochondrial volume or network complexity

**Fig. 1:**
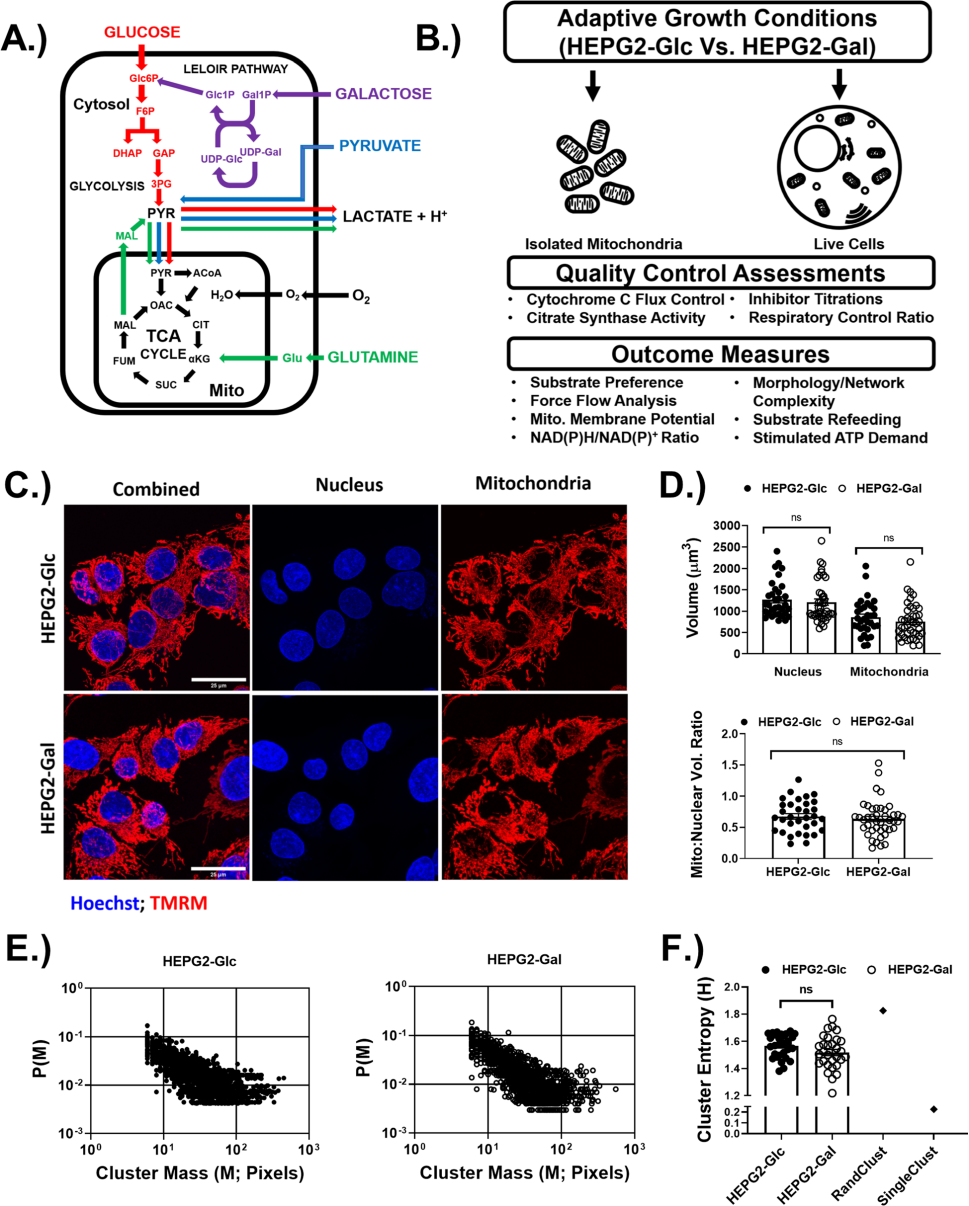
Aglycemic growth adaptation did not alter mitochondrial content or network complexity. **a** Metabolic pathway representation of carbon fuel sources relevant to the growth conditions used in this study. **b** Graphical summary of the experimental design. **c** Representative laser scanning confocal images of HEPG2 cells stained with nuclear counterstain (Hoechst) and mitochondria-localized dye (TMRM). **d** Nuclear and mitochondrial volumes and the ratios of mitochondrial to nuclear volumes. *N* = 30 cells/group—3 biological replicates. **e** Log scale probability of mitochondrial cluster masses P(M). *N* = 30 cells/group (**f**) Cluster entropy (**h**). RandClust indicates simulated maximum entropy value, SingleClust indicates minimum possible entropy value. *N* = 30 cells/group—3 biological replicates. Data are mean ± SEM. Means were compared using Student’s *t* test. *p < 0.05 HEPG2-Glc vs. HEPG2-Gal. Scale bars in images are 25 μm

Substitution of media glucose with galactose (aglycemic condition) most likely exerts its effects by limiting glucose availability to central carbon metabolism [50, 51]. Conversion of galactose to glucose through the Leloir pathway requires a uridine diphosphate glucose substrate pool for the galactose-1-phosphate uridyltransferase reaction, which is most likely the limiting reaction in this model due to the chronic absence of exogenous glucose (Fig. 1a). In the present study, we performed metabolic phenotyping in isolated mitochondria and live cells from high-galactose adapted (HEPG2-Gal) cells and compared several metabolic outcome measures with high-glucose adapted (HEPG2-Glc) control cells (Fig. 1b).

Adaptation to aglycemic growth conditions may lead to variation in mitochondrial volume, which would likely confound comparative metabolic measurements. To address this possibility, mitochondrial volume (as well as reference nuclear volume) was measured in both HEPG2-Gal and HEPG2-Glc cells. Cells from both growth conditions had comparable nuclear and mitochondrial volumes, as well as mito to nuclear volume ratios (Fig. 1c, d). Additionally, citrate synthase enzyme activity did not differ between isolated mitochondrial fractions from both growth conditions (SFigure 2A). Together, these findings indicate that mitochondrial content did not differ between the groups. Conversely, studies have indicated that environmental or genetically induced changes in ETS flux result in adaptive structural re-organization of the mitochondrial network [31, 52]. To investigate the possibility that similar morphological effects are induced under aglycemic growth conditions, we quantitated mitochondrial network complexity in live HEPG2-Glc and HEPG2-Gal cells using laser scanning confocal microscopy. The size distribution of connected mitochondrial clusters within samples of individual cells from both growth conditions was characterized by a relatively high probability (P(M)) of small clusters and a relatively low probability of large clusters (Fig. 1e). Mean cluster number and mass were similar among cells from both growth conditions (SFigure 1A–D). As a way of more directly summarizing network complexity for comparison, cluster entropy was also determined using an approach that is similar to a previously reported method [30]. A high-entropy value would indicate that cluster masses are uniformly distributed among their possible values (maximal value is RandClust) (Fig. 1f). A low entropy value would indicate that cluster masses are skewed toward a small number of possible values (minimal value indicated by SingleClust) (Fig. 1f). The observed cluster mass distributions were characterized by relatively high-entropy values indicating that cluster masses were spread out among their possible values, and no differences in cluster entropy were observed between cells from the two growth conditions, indicating a similar degree of network complexity (Fig. 1f).

### Aglycemic growth conditions do not enhance intrinsic mitochondrial oxidative capacity or efficiency

**Fig. 2:**
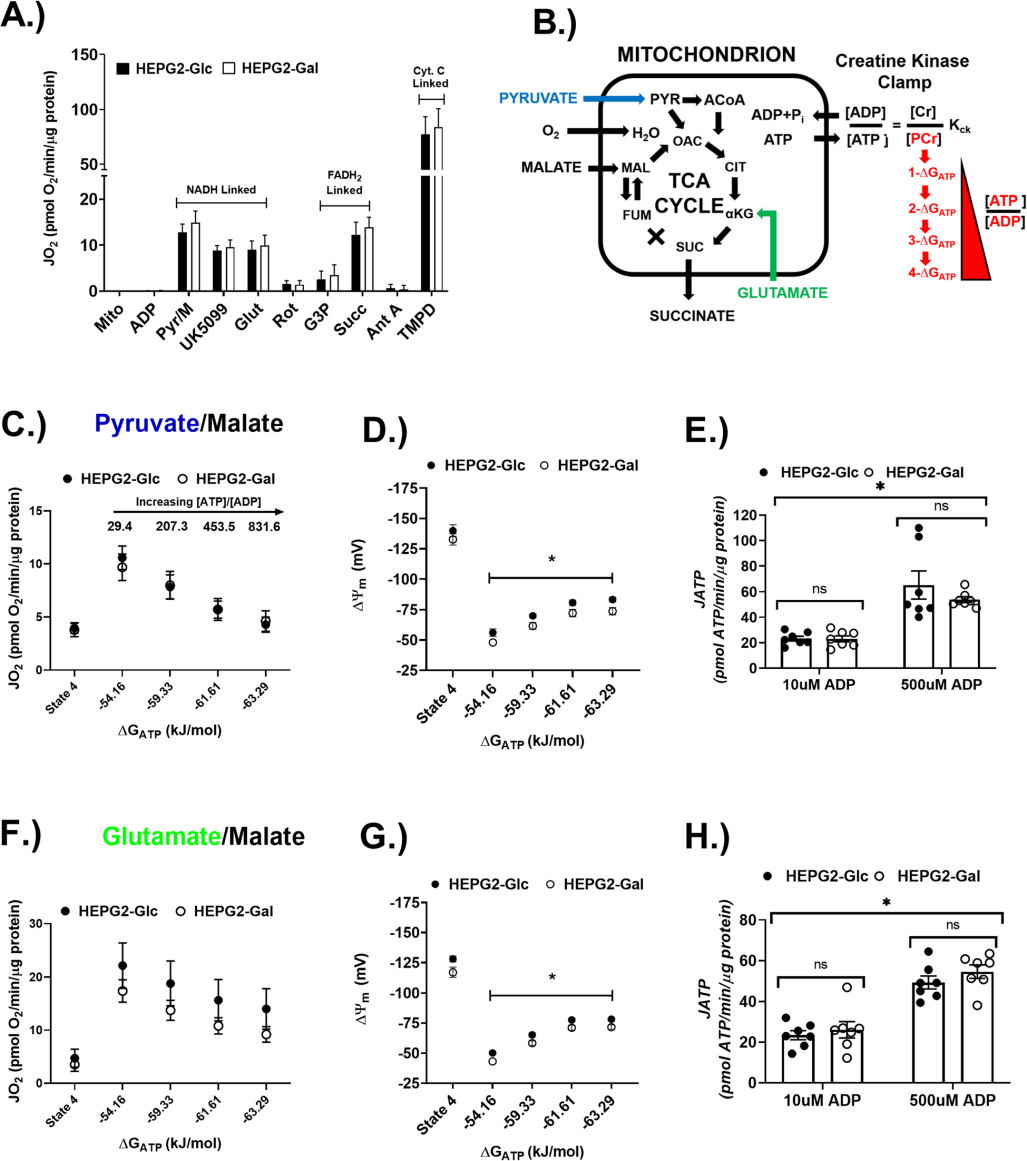
Intrinsic mitochondrial function was not affected by aglycemic growth. **a** Maximal respiratory capacities determined using substrate/inhibitor titration protocol in isolated mitochondria from HEPG2-Glc and HEPG2-Gal cells. ADP concentration was clamped at 300 μM using a hexokinase-coupled system. **b** Schematic of the creatine kinase free energy (∆G_ATP_) clamp system used in **c**, **d**, **f**, and **g**. **c** Pyruvate/malate-supported respiration over the clamped ∆G_ATP_ range. **d** Pyruvate/malate-supported electrical potential across the inner mitochondrial membrane (∆Ψ_m_) measured using tetramethyl rhodamine-methylester (TMRM) over the clamped ∆G_ATP_ range. **f** Pyruvate/malate-supported ATP production rate (*J*ATP) determined fluorometrically using a coupled hexokinase/glucose-6-phosphate dehydrogenase system under clamped ADP concentrations. **f**–**h** Glutamate/malate-supported *J*O_2_, (∆Ψ_m_), and *J*ATP. *N* = 7/treatment/group. Data are mean ± SEM. Means were compared using Student’s *t* test (**a**) and a two-way ANOVA with Sidak’s multiple comparison test (**c**–**h**). *p < 0.05

HEPG2 cells grown in galactose have been previously described as “aerobically poised,” characterized by enhanced basal rate of oxygen consumption and decreased extracellular acidification rate [6, 17, 18]. However, it is unclear if galactose induces adaptations in intrinsic mitochondrial function or other, more complex whole-cell adaptations. To address this question, mitochondria were isolated from HEPG2-Glc and HEPG2-Gal cells and subjected to detailed in vitro phenotyping of mitochondrial function. Mean citrate synthase activity did not differ between mitochondrial preparations isolated from either growth condition, indicating that the preparations had similar purity (SFigure 2A). Maximal NADH-, FADH_2_-, or CytC-linked ADP-stimulated respiration did not differ in mitochondria isolated from the HEPG2 cells grown under the two different conditions (Fig. 2a). Respiration and electrical potential across the inner mitochondrial membrane (ΔΨ_m_) were assessed in a manner that more closely models the oxidative phosphorylation (OxPhos) system in vivo by clamping extra-mitochondrial ATP/ADP ratios (i.e., the free energy of ATP hydrolysis; ΔG_ATP_) over a broad range of respiratory demand states (Fig. 2b). *J*O_2_ responses were not different between HEPG2-Glc and HEPG2-Gal mitochondria during respiration supported by pyruvate/malate (Fig. 2c, d) or glutamate/malate (Fig. 2f, g). Interestingly, HEPG2-Glc mitochondria were slightly more polarized (i.e., higher ΔΨ_m_ values) than HEPG2-Gal mitochondria over the respiratory demand range under both fuel conditions (Fig. 2d, g). ATP production rates (*J*ATP) were assessed using a hexokinase clamp system [41] at two different steady-state rates of ADP-stimulated respiration. *J*ATP was not different between mitochondria from cells grown in glucose versus galactose under either pyruvate/malate (Fig. 2e) or glutamate/malate (Fig. 2h) supported conditions. ATP/O stoichiometric ratios were also not different under any of the tested conditions (Table 1). Finally, NAD(P)^+^/NAD(P)H autofluorescence, a parameter reflecting the redox state driven by matrix dehydrogenase enzymes, also did not differ between growth conditions over the same range of ΔG_ATP_ (SFigure 2B,C). Given the similarities in indices of fuel supply (NAD(P)^+^/NAD(P)H) and OxPhos efficiency (*J*ATP and ATP/O) between growth conditions, the origin and biological significance of the slightly lower ΔΨ_m_ in mitochondria from cells grown in galactose is unclear.

### Aglycemic growth conditions result in adaptive changes to glucose metabolism

To further explore basal metabolic adaptations to aglycemic growth, oxygen consumption and acidification rates were measured in intact cells. To account for non-respiratory oxygen consumption rate (*J*O_2_), measured rates were corrected to those obtained in the presence of antimycin A (cytochrome bc_1_ complex inhibitor) plus rotenone (NADH oxidase inhibitor). Additionally, extracellular acidification rates (ECAR) were converted to more useful units (proton efflux rate; *J*H^+^) using empirically derived buffer capacity measurements. Finally, compartmentalized flux patterns through central carbon metabolism were determined by separately refeeding exogenous glucose, pyruvate, or glutamine. This strategy was combined with the use of inhibitors to limit mitochondria-specific respiration and induce compensatory flux through glycolysis.

**Fig. 3:**
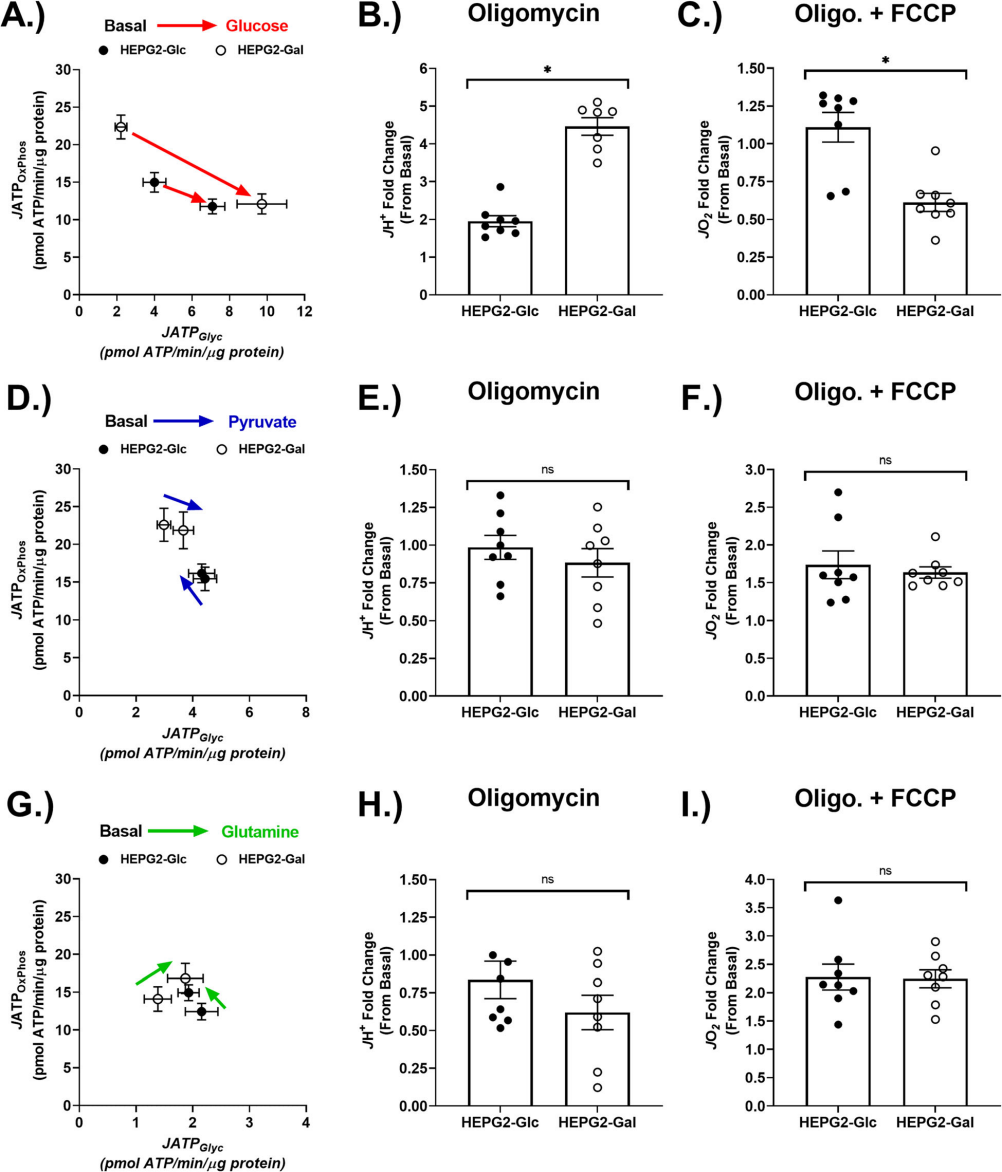
Adaptation to aglycemic growth conditions enhanced glucose-supported energy partitioning in HEPG2 cells. **a** Bivariate plot of estimated energy partitioning between oxidative phosphorylation (*J*ATP_OxPhos_) and glycolysis (*J*ATP_Glyc_) in response to glucose refeeding. **b** Glucose-supported proton efflux rate (*J*H^+^) fold change between basal condition (prior to glucose refeeding) and a high glycolytic flux condition: induced using 5 μM oligomycin. **c** Glucose-supported respiration rate (*J*O_2_) fold change between basal and a high respiratory flux condition (5 μM oligomycin + 1 μM FCCP). **d** Estimated *J*ATP in response to pyruvate refeeding. **e** Pyruvate-supported *J*H^+^ under a high glycolytic flux condition (5 μM oligomycin). **f** Pyruvate-supported *J*O_2_ fold change between basal and a high respiratory flux condition (5 μM oligomycin + 1 μM FCCP). **g** Estimated *J*ATP in response to glutamine refeeding. **h** Glutamine-supported *J*H^+^ under a high glycolytic flux condition (5 μM oligomycin). **i** Glutamine-supported *J*O_2_ fold change between basal and a high respiratory flux condition (5 μM oligomycin + 1 μM FCCP). *N* = 8/treatment/group. Data are mean ± SEM. Means were compared using Student’s *t* test or two-way ANOVA (**c**). *p < 0.05. ns = not significant

Glycolysis produces net ATP equivalents via substrate-level phosphorylation coupled with pyruvate fermentation to lactate and/or via oxidative phosphorylation coupled with the oxidation of pyruvate in the TCA cycle (Fig. 1a). *J*ATP was estimated by combining empirical measurements with stoichiometric coefficients (Table 2) as previously described [44]. Notably, the pattern of estimated *J*ATP partitioning between fermentation and OxPhos differed substantially between HEPG2-Glc and HEPG2-Gal cells following glucose refeeding (Fig. 3a). However, total *J*ATP did not differ between the two growth conditions, indicating a similar rate of metabolic demand (SFigure 3A). Both HEPG2-Glc and HEPG2-Gal cells exhibited increased *J*H^+^ and decreased *J*O_2_ in response to glucose refeeding (SFigure 3A, B); however, this effect was more pronounced in HEPG2-Gal cells. HEPG2-Gal cells also exhibited a greater compensatory increase in *J*H^+^ in response to oligomycin (Fig. 3b, SFigure 3C). Interestingly, this effect was accompanied by a substantial reduction in maximal uncoupled respiration (relative to basal), indicating that mitochondrial respiration is repressed in response to glucose refeeding (Fig. 3c, SFigure 3D).

**Table 2:** Stoichiometric constants and correction factors used for intact cell *J*ATP estimation

Substrate (S)	Max O/mol S	Max H^+^ pumped/mol S	ATP from SCS	Total ATP (mol S^−1^)	P/O (total ATP/mol O)
d-Glucose - > CO_2_ (MAS active)	12	110	2	33.45	2.78
Pyruvate - > CO_2_	5	46	1	13.27	2.654
Glutamine - > CO_2_ (pyruvate cycle active)	8	62	2	18.9	2.36

ATP/O ratios for substrate refeeding conditions were estimated as previously described [37]. The following values were assumed: 11/3 H^+^/ATP phosphorylated; 2e^-^/O; 2NADH (or 2QH_2_)/O_2_; 1ATP/GTP at succinyl CoA synthetase (SCS). Malate aspartate shuttle (MAS)

Exogenous pyruvate can be reduced to lactate by cytosolic lactate dehydrogenase or oxidized in the TCA cycle; however, net ATP equivalents are only produced in the latter instance (Fig. 1a). The pattern of estimated *J*ATP partitioning between fermentation and OxPhos did not differ substantially between HEPG2-Glc and HEPG2-Gal cells following pyruvate refeeding (Fig. 3d). Total *J*ATP did not differ between the two growth conditions, again indicating a similar rate of metabolic demand (SFigure 3D). HEPG2-Gal cells exhibited no substantial changes in *J*H^+^ or *J*O_2_ in response to pyruvate refeeding (SFigure 3E). No compensatory increases in *J*H^+^ (from basal) in response to oligomycin were observed in either HEPG2-Glc or HEPG2-Gal cells (Fig. 3e, SFigure 3F), nor were any differences in maximal uncoupled respiration detected between the two growth conditions (Fig. 3f, SFigure 3F).

Exogenous glutamine contributes anaplerotic substrates to the TCA cycle through glutaminolysis. Under normal circumstances, glutamine-derived alpha-ketoglutarate maintains the oxaloacetate pool, but in the absence of exogenous sources of pyruvate, glutamine is able to support replenishment of oxaloacetate and acetyl-CoA through pyruvate (Fig. 1a) [53]. The pattern of estimated *J*ATP partitioning between fermentation and OxPhos did not differ between HEPG2-Glc and HEPG2-Gal cells following glutamine refeeding (Fig. 3g). Total *J*ATP also did not differ between growth conditions (SFigure 3G). HEPG2-Gal cells exhibited no differences in *J*H^+^ or *J*O_2_ in response to glutamine refeeding (SFigure 3H). No compensatory increases in *J*H^+^ (from basal) in response to oligomycin were observed in both HEPG2-Glc and HEPG2-Gal cells (Fig. 3h, SFigure 3H) nor were any differences in maximal uncoupled respiration detected between the two growth conditions (Fig. 3i, SFigure 3I).

Interestingly, in the presence of respiratory complex I and III inhibitors rotenone and antimycin A, *J*O_2_ was higher in HEPG2-Gal cells, indicating that oxidase activity not associated with the electron transfer system was enhanced (SFigure 4A). Oligomycin rates (corrected for rotenone/antimycin A) did not differ, indicating that proton “leak” rates were not altered by aglycemic growth adaptation (SFigure 4B). Finally, the apparent coupling coefficient (Q), an indicator of the fraction of respiration associated with ATP oxidative phosphorylation, did differ between substrate refeeding conditions (highest with glutamine) but did not differ between cells from either growth condition (SFigure 4C).

**Fig. 4:**
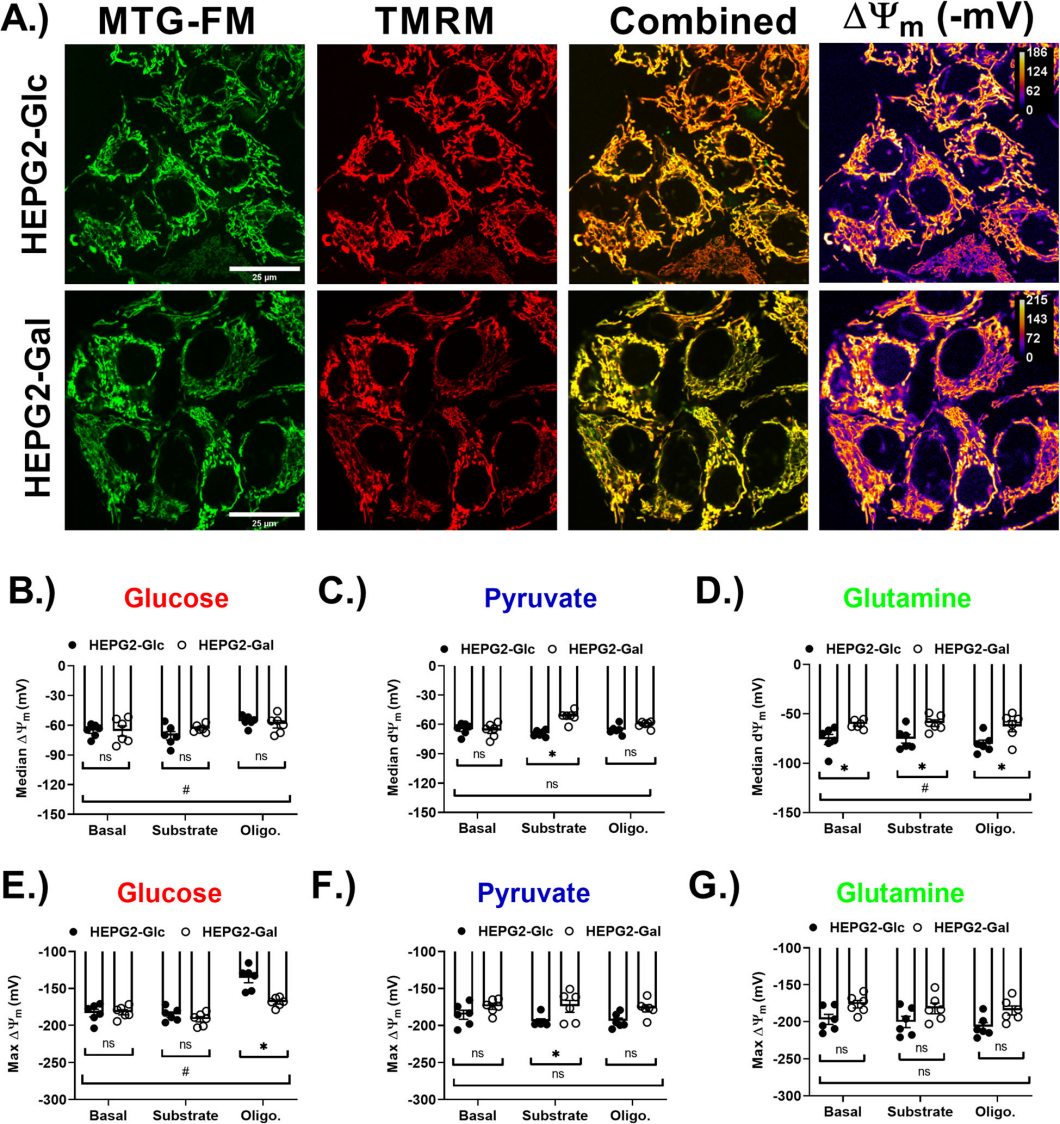
Electrical potential across the inner mitochondrial membrane (ΔΨ_m_) was sensitive to oligomycin following glucose refeeding. **a** Representative LSCM images of HEPG2-Glc and HEPG2-Gal dual-stained with mitotracker green-FM (MTG-FM) and non-quenching tetramethylrhodamine methylester (TMRM). ΔΨ_m_ values were determined using measured TMRM intensities and were mapped onto images using the math macro in ImageJ. **b** Median ΔΨ_m_ values obtained from 3D stacks of HEPG2-Glc and HEPG2-Gal cells with glucose refeeding (25 mM) followed by inhibition of the F_1_F_o_ ATPase with oligomycin A. **c** Median ΔΨ_m_ values obtained from 3D stacks of HEPG2-Glc and HEPG2-Gal cells with pyruvate refeeding (1 mM). **d** Median ΔΨ_m_ values obtained from 3D stacks of HEPG2-Glc and HEPG2-Gal cells with glutamine refeeding (4 mM). **e** Median ΔΨ_m_ values obtained from 3D stacks of HEPG2-Glc and HEPG2-Gal cells with glucose refeeding (25 mM) followed by inhibition of the F_1_F_o_ ATPase with oligomycin A. **f** Median ΔΨ_m_ values obtained from 3D stacks of HEPG2-Glc and HEPG2-Gal cells with pyruvate refeeding (1 mM). **g** Median ΔΨ_m_ values obtained from 3D stacks of HEPG2-Glc and HEPG2-Gal cells with glutamine refeeding (4 mM). *N* = 6/treatment/group. Data are mean ± SEM. Means were compared using a two-way ANOVA. Scale bars are 25 μm

Mitochondrial membrane potential (ΔΨ_m_) measurements in isolated preparations are limited by the necessity of adding substrates at saturating concentrations to maintain the respiratory steady-state [54]. To investigate differences in ΔΨ_m_ in situ, live intact cells were stained with the potentiometric dye tetramethyl rhodamine methyl ester (TMRM) and the non-potentiometric carbocyanine dye mitotracker green-FM (as a total mitochondrial counterstain) and imaged under substrate/inhibitor conditions that matched the substrate refeeding experiments (Fig. 3). Heterogeneity in ΔΨ_m_ values within individual cells was observed in both growth conditions (Fig. 4a), so ΔΨ_m_ measurements are represented with both median and maximal values for each condition. Notably, median and maximum ΔΨ_m_ values among the groups were similar, which was interpreted to mean that the degree of heterogeneity was not influenced by growth condition. Median ΔΨ_m_ was significantly lower in HEPG2-Gal cells following addition of either pyruvate or glutamine, but not glucose (Fig. 4b–d). Interestingly, refeeding with glucose resulted in a relatively large decrease in HEPG2-Gal maximal ΔΨ_m_ upon addition of oligomycin (~ 30% drop in HEPG2-Gal vs. ~ 10% drop in HEPG2-Glc) (Fig. 4e). This observation is consistent with the attenuated response in uncoupled (FCCP) respiration during the extracellular flux analysis (EFA) experiments (Fig. 3e). Maximal ΔΨ_m_ values were similar among the two cell lines for the pyruvate and glutamine refeeding conditions (Fig. 4f, g). An additional image panel is available in the online supplement that confirms the expected decrease in intra-mitochondrial fluorescence intensity following depolarization of ΔΨ_m_ by addition of FCCP (SFigure 5).

### Aglycemic growth conditions induce adaptation of demand-stimulated metabolic rates

**Fig. 5:**
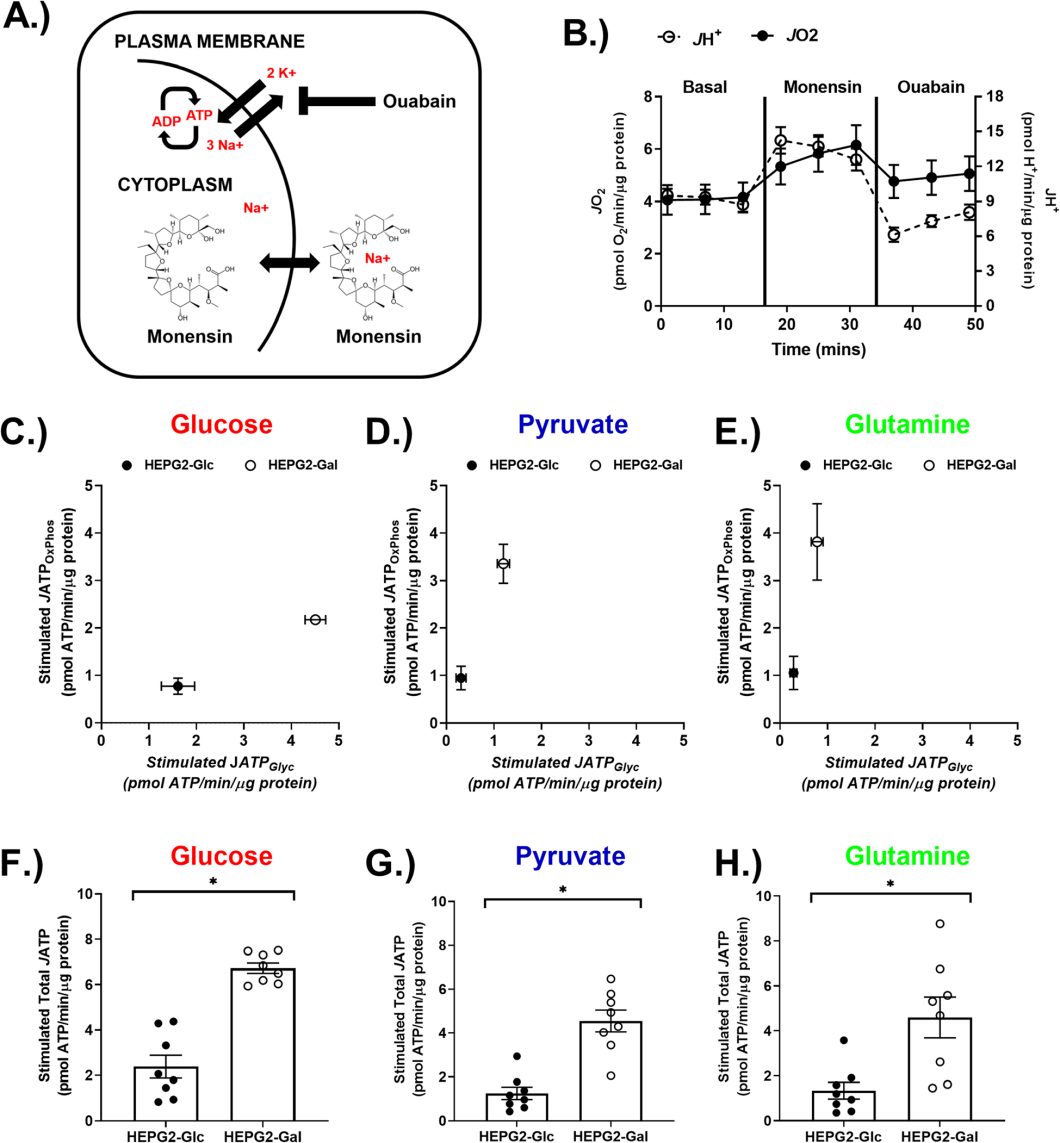
Adaptation to aglycemic growth conditions enhanced metabolic response to plasmalemmal sodium potential uncoupling. To assess the response to an increased energy demand, the cells were exposed to the sodium ionophore monensin, which induces increased ATP utilization through Na^+^/K^+^ ATPase pumps that is sensitive to the Na^+^/K^+^ ATPase inhibitor ouabain. **a** Schematic diagram of monensin action. **b** Example trace of respiration (*J*O_2_) and proton efflux (*J*H^+^) rates in response to monensin, followed by inhibition of the Na^+^/K^+^ ATPase inhibitor ouabain. **c** Glucose-supported stimulated energy demand (calculated by subtracting the basal rate from the ouabain-corrected monensin rate for both *J*ATP_OxPhos_ and *J*ATP_Glyc_) represents the rate *increase* that is attributable to the monensin. **d** Pyruvate-supported stimulated energy demand. **e** Glutamine-supported stimulated energy demand. **f** Glucose-supported total stimulated ATP production rates (sum of OxPhos and glycolysis). **g** Pyruvate-supported total stimulated ATP production rates. **h** Glutamine-supported total stimulated ATP production rates. *N* = 8/treatment/group. Data are mean ± SEM. Means were compared using Student’s *t* test. *p < 0.05. ns = not significant

To stimulate energetic demand and therefore metabolic flux, the sodium ionophore monensin [47] was added to partially depolarize the plasma membrane sodium potential, which results in an energy-depleting cycle that is dependent on the Na^+^/K^+^ ATPase (Fig. 5a, b). Stimulated *J*ATP was quantitated using the monensin-induced *J*O_2_ and *J*H^+^ rate increases minus rates measured following subsequent addition of ouabain to inhibit the Na^+^/K^+^ ATPase. HEPG2-Gal cells exhibited enhanced stimulated *J*ATP through both OxPhos and, to a lesser extent, glycolysis (Fig. 5c–e). Greater total *J*ATP supported by all three substrate conditions was also observed (Fig. 5f–h).

### Aglycemic growth conditions result in selective sensitivity to the redox cycling agent menadione

**Fig. 6:**
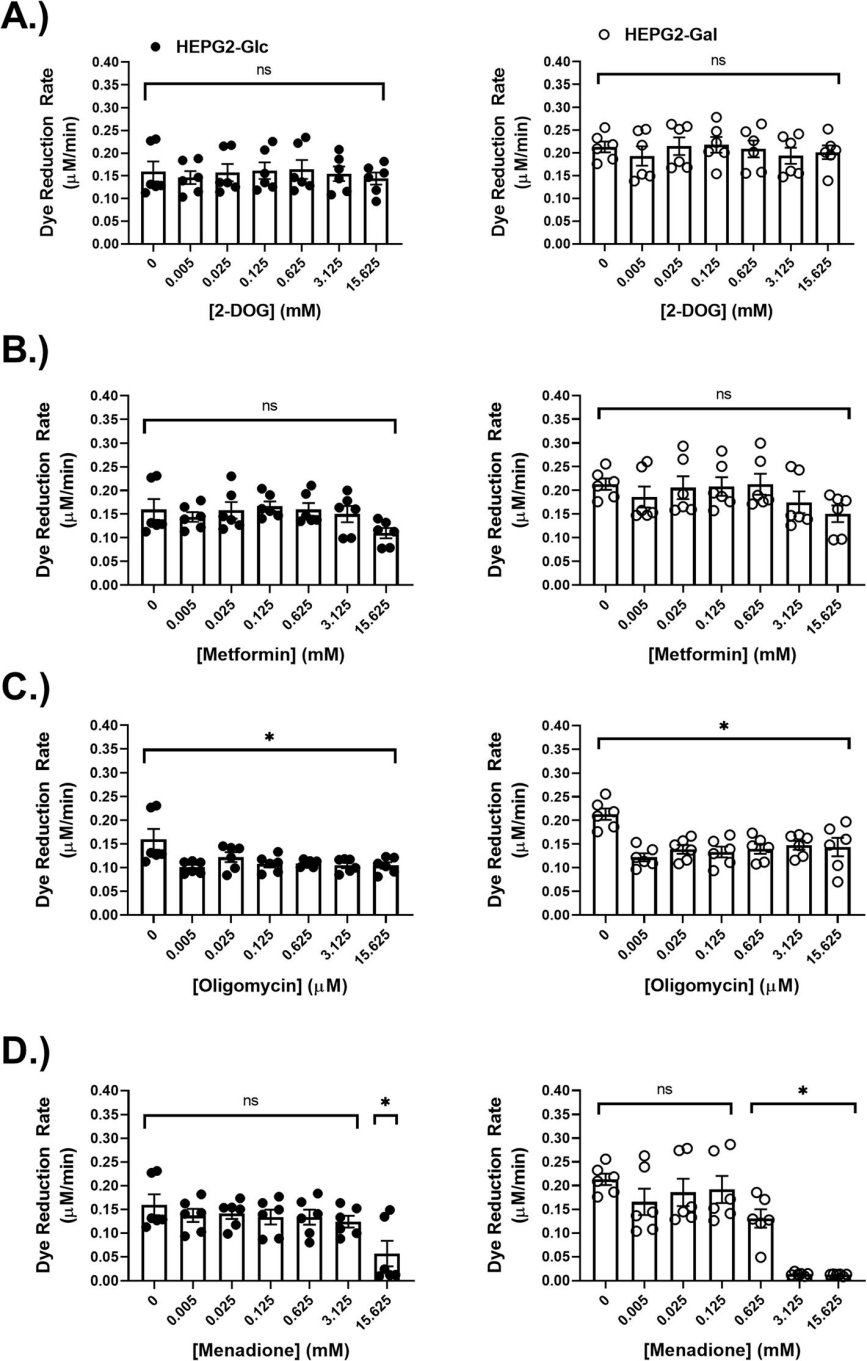
Aglycemic growth adaptation induced sensitivity to redox **c**ycling agent menadione. **a** Resazurin dye reduction rates (as an indicator of cell viability) for cells exposed to 2-deoxy-glucose (2-DOG) for 24 h in DMEM-L-GLC media. **b** Complex I inhibitor—metformin-treated cells (dimethyl biguanide). **c** F_o_F_1_ ATPase inhibitor—oligomycin-treated cells. **d** Redox cycling agent—menadione-treated cells. *N* = 6 wells/treatment/group. Data are mean ± SEM. Means were compared using one-way ANOVA with multiple comparisons against compound-free control cells

To determine whether aglycemic growth adaptation causes specific sensitivities to cytotoxic agents or changes in nutrient availability (as would occur during IOX implantation), cells were switched to a media designed to more closely approximate substrate concentrations found in serum [55] (DMEM-L-GLC) and were incubated for 24 h with five-fold serial dilutions of compounds that target metabolic features in distinct ways. 2-Deoxy-glucose (2-DOG) is a glucose antimetabolite, and its accumulation results in osmotic stress and glycolysis inhibition [11]. Metformin (dimethyl biguanide) is an organic cation that acts as an inhibitor of respiratory complex I at micromolar concentrations [56–58]. The macrolide antibiotic oligomycin is a potent inhibitor of the F_o_F_1_ ATP synthase [18]. Menadione is a vitamin K analog that participates in a redox cycle with redox active enzymes resulting in substantial production of reactive oxygen species [59]. Neither 2-DOG nor metformin had any discernable effect on cell viability (measured via resazurin dye reduction rate) in cells from either growth condition (Fig. 6a–d). Oligomycin reduced viability similarly in cells from both growth conditions across its entire dose range (Fig. 6e, f). Notably, HEPG2-Gal cells were more sensitive to high concentrations of menadione compared to HEPG2-Glc cells (Fig. 6g, h).

## Discussion

### Galactose substitution as a model for aglycemic growth conditions

Substitution of media glucose for galactose as a model of aglycemic growth has been used for decades to investigate limitation of carbohydrate metabolism in cultured cells [15, 18, 50]. Cells grown in galactose have been previously described as “aerobically poised,” characterized by enhanced basal rate of oxygen consumption and decreased extracellular acidification rate [6, 17, 18]. However, the exact metabolic adaptations, particularly with respect to partitioning of ATP production between substrate-level phosphorylation in glycolysis and mitochondrial oxidative phosphorylation have not been well defined. Galactose growth conditions are often described as imposing a stoichiometric ATP constraint on glycolytic ATP production [17, 18, 60, 61]. In this model description, galactose conversion to galactose-1-phosphate by galactokinase requires one ATP molecule. Glucose-1-phosphate is produced from galactose-1-phosphate by the galactose-1-uridyl transferase reaction at the expense of UDP-glucose, which is converted to UDP-galactose. The resulting glucose-1-phosphate is then converted to glucose-6-phosphate by phosphoglucomutase. UDP-glucose is synthesized from glucose-1-phosphate by UDP-glucose pyrophosphorylase. This reaction requires UTP, which is considered an ATP equivalent. Because this pathway costs two net equivalents of ATP, flux through glycolysis that is solely dependent on glucose-6-phosphate derived from galactolysis would yield no net ATP equivalents [60, 61].

However, the model described above has not been definitively tested and studies that used radio-isotope-labeled carbohydrates in cultured cells found that galactose carbons did not enter glycolysis at an appreciable rate [50, 51, 62]. More likely, the galactose-1-uridyl transferase reaction is limited by the size of the UDP-glucose pool, which is restricted because the cells are starved of exogenous glucose [63]. This suggests that galactose growth conditions more closely simulate a state of glucose deprivation, rather than stoichiometric negation of net glycolytic ATP production. However, the model is still advantageous because it facilitates comparison of carbohydrate-restricted growth conditions under matched media osmotic pressures and provides a minimal carbohydrate source on which many cell lines can maintain growth rates that are comparable to glucose-supported conditions [64].

### Cellular energetic adaptations to aglycemic growth conditions

HEPG2 cells are favored for tumor-derived cell metabolism studies due to their apparent metabolic flexibility and pseudo-differentiated phenotype [19–22]. When challenged with restricted nutrient conditions, HEPG2 cells are capable of rapidly switching between a balanced state of combined oxidative energy metabolism and aerobic fermentation to a state dominated by only one of these elementary modes [19, 25, 26, 60, 61]. When grown in aglycemic conditions, HEPG2 cells develop sensitivity to toxicants that affect mitochondrial function, which has been used as a useful tool for identifying drugs with mitochondrial liabilities [6, 17, 18]. The interpretation of these observations has been that aglycemic growth conditions result in an obligate shift from a primary reliance on glycolytic substrate-level phosphorylation to mitochondrial oxidative phosphorylation for energy metabolism (OxPhos) [18, 25, 60].

The findings in this study do not strictly support the phenomenon of adaptive “aerobic poise.” Though HEPG2 cells did thrive under aglycemic conditions (likely by oxidizing exogenous glutamine and pyruvate among other trace media fuel sources), they did not enhance their intrinsic capacity or efficiency to utilize these fuel sources, which was assessed over a range of physiologically relevant demand states in isolated mitochondria [37, 40]. A slight increase in basal respiratory rate independent of mitochondrial content or network complexity was observed in live cells, which is consistent with other reports [6, 18, 64]. However, the most notable adaptive response to aglycemic growth observed was in the apportioning of energy metabolism between substrate-level phosphorylation (glycolytic fermentation) and oxidative phosphorylation following refeeding with glucose, supporting the conclusion that carbohydrate metabolism is the primary target of adaptation in this model. Together, these observations showcase the importance of combining both isolated mitochondria and live cell metabolic flux measurements and highlight the importance of interpreting either set of measurements cautiously (if performed on their own).

There are several possible underlying mechanisms by which the rate of carbohydrate metabolism could be enhanced. A recent study that used metabolic flux analysis in combination with selective overexpression of glycolytic pathway enzymes in immortalized mouse kidney cells determined that control over glycolytic flux was largely attributable to only four steps: glucose transport, hexokinase, phosphofructokinase, and lactic acid efflux (through monocarboxylate transporters) [65]. Adaptive changes in activity or expression of one or more of these enzymes may have contributed to the observations in the present study.

Interestingly, glucose refeeding rapidly repressed the rate of respiration in HEPG2-Gal cells, while simultaneously increasing *J*H^+^, a phenomenon that resembles the Crabtree effect in yeast [66]. There are two likely explanations for these observations. The first is that the increase in ADP phosphorylation rate by substrate-level phosphorylation inhibited OxPhos through respiratory control exerted via the free energy of the ATP hydrolysis reaction (similar to kinetic effects demonstrated in the force-flow experiments) [38, 40, 67]. This is further supported by the similarity in the estimated total *J*ATP between groups, indicating that glucose refeeding induced a redistribution of flux through energy-transducing pathways. Alternatively, the increased rate of pyruvate reduction to lactic acid may have simply outpaced the transport/oxidation of pyruvate in the mitochondria. However, exogenous pyruvate refeeding did not substantially increase *J*H^+^ suggesting that the first explanation is more likely.

Another interesting observation was that rotenone/antimycin A-insensitive oxygen consumption rate was greater in HEPG2-Gal compared with HEPG2-Glc cells. This was interpreted to represent enhanced expression or activity of oxidase enzymes that are not associated with the electron transfer system. Though the enzymes underlying the putative oxidase activity were not identified in this study, there are some plausible candidates that should be investigated in future studies. First, NAD(P)H oxidase (NOX) enzyme family members are expressed in HEPG2 cells and have been implicated in regulation of central carbon metabolism [68]. However, it has also been previously reported that NOX family member expression/activity is enhanced under high- (rather than low) glycemic conditions [60]. Alternatively, the Cytochrome P450 family of monooxygenases are also expressed in HEPG2 cells, but their expression is generally considered to be low [69]. However, increased expression/activity may occur in response to aglycemic conditions [70].

Though the free energy clamp used for the isolated mitochondria experiments facilitated titration of the energetic demand state over a physiologically relevant range [37, 40], there is no precise method to do this in live cells. However, the sodium ionophore monensin can be used to stimulate an energetic demand by depolarizing the plasma membrane sodium potential [47]. Glucose supported the highest stimulated rate of ATP production (primarily through aerobic fermentation). This finding suggests that glycolytically derived ATP responds to fluctuations in peak cellular energetic demand (e.g., plasma membrane potential maintenance), while OxPhos-derived ATP supports basal energetic demands (e.g., macromolecular synthesis), which is congruent with a previous report that used a similar method [71]. It is unclear why HEPG2-Gal cells exhibited greater stimulated *J*ATP in response to monensin treatment. Notably, it has been reported that HEPG2 cells are more sensitive to ouabain treatment under glucose-deprived conditions [72]. This suggests that Na^+^/K^+^ ATPase expression/activity is important for adaptation to aglycemia and may be upregulated under these conditions. Increased Na^+^/K^+^ ATPase expression/activity would be expected to increase the total ATP demand in response to monensin, which may account for the observed effects.

### Relevance to the IOX model

Intrahepatic orthotopic xenograft (IOX) models in which primary or tumor-derived cell lines are implanted into rodent livers have been used to predict drug efficacy and provide information about metastatic patterns [8]. Due to cell line heterogeneity, use of multiple cell lines is recommended for drug screening in order to account for phenotypic variability [8]. This report provides a detailed description of adaptive variation in metabolic phenotype induced by modification of macronutrient sources in growth medium. This may have some useful implications for the design of IOX testing models; particularly, the potential impact of growth media conditions should be carefully considered, especially when multiple cell lines are used [16, 25, 26]. The phenotyping methods described in this study (e.g., substrate refeeding) may be useful in identifying metabolic implications associated with greater tumorgenicity, metastasis, or drug sensitivity, given that the observed adaptive effects were induced in cells from the same line that were grown in otherwise identical culture media (with the exception of carbohydrate source).

Previous studies have indicated that HEPG2 cells incubated in galactose growth media are more sensitive to staurosporine-induced apoptosis [60, 61], and several studies have shown specific sensitivities to many common drugs in cells cultured in galactose or other nutrient-limited media [6, 17, 18, 57]. However, the common practice in nutrient sensitization studies is to perform the cytotoxicity assays directly in the aglycemic- or nutrient-deprived media. To our knowledge, this is the only such report that has performed the cytotoxicity assays in normo-glycemic media following adaptation to aglycemic growth conditions. Of the four metabolic toxins assessed, only menadione elicited specific sensitivity in HEPG2-Gal cells. Combined with the observation that background oxidase activity was elevated, this may allude to a redox stress liability that is uncovered by the adaptive growth response. Menadione is a redox cycling agent, and its toxicity is enhanced by the activity/expression of oxidoreductase enzymes; thus, increased oxidase expression/activity may sensitize the cells to menadione [59]. Notably, another recent study found that HEPG2 cells treated with competitive inhibitors of glycolytic flux exhibited increased redox stress when treated with doxorubicin, further supporting a link between aglycemic growth and redox stress liability in these cells [73]. The relevance of such observations to cancer chemotherapeutic sensitivity requires further investigation, but is an attractive hypothesis due to the tendency of other common chemotherapeutics, particularly the HCC therapeutic sorafenib, to induce oxidative stress [2, 74–77].

## Conclusions

This study provides a detailed, multilevel systems approach to define specific bioenergetic adaptations to aglycemic growth conditions in HEPG2 cells. The approach involves parallel assessment at the organelle and whole-cell levels, structural and functional measurements, selective substrate refeeding to target specific modes of central carbon metabolism, and comparison of metabolic fluxes under different states of energetic demand. The hypothesis that aglycemic growth conditions would facilitate compensatory enhancement of oxidative metabolism and repression of aerobic fermentative metabolism did not strictly hold, as the results indicated that oxidative metabolism did not differ substantially between the two tested cell lines. However, fermentative substrate-level phosphorylation was substantially enhanced and a degree of selective sensitivity toward menadione toxicity was observed. These findings will support further hypothesis development, advance the understanding of implicit regulation of metabolic adaptation in tumor-derived cells, and improve IOX testing panels by providing a practical workflow that reports detailed information regarding the effects of subculture conditions on adaptive energy partitioning in tumor-derived cells.

## Supplementary Information

**Additional file 1: Supp. Figure 1**: Additional intact cell mitochondrial morphology data. (A) Image panel diagram of steps involved in identifying individual mitochondrial cluster distributions in individual cells from laser scanning confocal images of TMRM staining. (B) Image subpanel highlighting the qualitative differences in clusters with either small (S; purple), medium (M; red), or large (L; orange) cluster masses. *N* represents the nucleus. (C) Mean cluster number (per cell). *N* = 30 cells/group (D) Mean cluster mass. *N* = 30 cells/group. Data are mean ± SEM. Means were compared using Student’s *t* test. Data are mean ± SEM. *p < 0.05. ns = not significant.

**Additional file 2: Supp. Figure 2**: Additional isolated mitochondria data. (A) Citrate synthase activity in mitochondria isolated from HEPG2-Glc and HEPG2-Gal cells. (B) NAD(P)^+^/NAD(P)H autofluorescence is proportional to the redox state of matrix dehydrogenase reactions for pyruvate/malate-supported respiration. (C) NAD(P)^+^/NAD(P)H autofluorescence is proportional to the redox state of matrix dehydrogenase reactions for glutamate/malate-supported respiration. Data are represented as a percent of the fluorescence measured in the presence of potassium cyanide (10 mM). *N* = 7/treatment/group. Data are mean ± SEM. Means were compared using Student’s *t* test (A) and a two-way ANOVA with Sidak’s multiple comparison test (B, C). *p < 0.05.

**Additional file 3: Supp. Figure 3**: Additional intact cell respiration data and total *J*ATP values: (A) Estimated total ATP production rates (*J*ATP) attributable to both OxPhos and aerobic fermentation at baseline and following glucose refeeding. (B) Bivariate plot of respiration rate (*J*O_2_) vs. proton efflux rate (*J*H^+^) at baseline and following glucose refeeding. (C) Bivariate plot of glucose-supported *J*O_2_ vs. *J*H^+^ during a high glycolytic flux condition (5 μM Oligomycin) and a high respiratory flux condition (5 μM Oligomycin + 1 μM FCCP). (D) Estimated total ATP production rates (*J*ATP) attributable to both OxPhos and aerobic fermentation at baseline and following pyruvate refeeding. (E) Respiration rate (*J*O_2_) vs. proton efflux rate (*J*H^+^) at baseline and following pyruvate refeeding. (F) Pyruvate-supported *J*O_2_ vs. *J*H^+^ during a high glycolytic flux condition and a high respiratory flux condition. (G) Estimated total ATP production rates (*J*ATP) attributable to both OxPhos and aerobic fermentation at baseline and following pyruvate refeeding. (H) Respiration rate (*J*O_2_) vs. proton efflux rate (*J*H^+^) at baseline and following pyruvate refeeding. (I) Pyruvate-supported *J*O_2_ vs. *J*H^+^ during a high glycolytic flux condition and a high respiratory flux condition. Data are mean ± SEM. Means were compared using a two-way ANOVA (A, D, G). *N* = 8/treatment/group. *p < 0.05. ns = not significant.

**Additional file 4: Supp. Figure 4**: Additional intact cell inhibitor data and apparent coupling efficiencies: (A) *J*O_2_ following exposure to the NADH oxidoreductase inhibitor rotenone and cytochrome bc1 complex inhibitor antimycin A. (B) *J*O_2_ following exposure to F_o_F_1_ ATPase inhibitor oligomycin. (C) Apparent coupling efficiencies (Q) for each substrate determined from the fractional change in respiration that occurred following inhibition by oligomycin. Data are mean ± SEM. *N* = 8/treatment/group. Means were compared using a two-way ANOVA with Sidak’s multiple comparison test. *p < 0.05. ns = not significant.

**Additional file 5: Supp. Figure 5**: Additional intact cell ΔΨ_m_ data: Image panel demonstrating the expected redistribution of mitochondrial localized dyes and reduction of ΔΨ_m_ in the presence of the cell permeable protonophore (Trifluoromethoxy carbonylcyanide phenylhydrazone; FCCP). Scale bars are 25 μm).

## Data Availability

The datasets generated and/or analyzed during the current study are available in the Open Science Framework (OSF) repository (DOI available on acceptance for publication).

## Abbreviations

HCC: Hepatocellular carcinoma IOX Intrahepatic orthotopic xenograft model TMRM Tetramethylrhodamine methyl ester *J*O_2_ Oxygen consumption rate ECAR Extracellular acidification rate EFA Extracellular flux analysis *J*H^+^ Proton efflux rate CK Creatine kinase CytC Cytochrome C PCr Phosphocreatine ATP Adenosine triphosphate
