# Supplementary figures and images for: Aglycemic growth enhances carbohydrate metabolism and induces sensitivity to menadione in cultured tumor-derived cells
Source: Cancer Metab. 2021 Jan 19;9:3. doi: 10.1186/s40170-021-00241-0 (PMC7816515; doi:10.1186/s40170-021-00241-0)

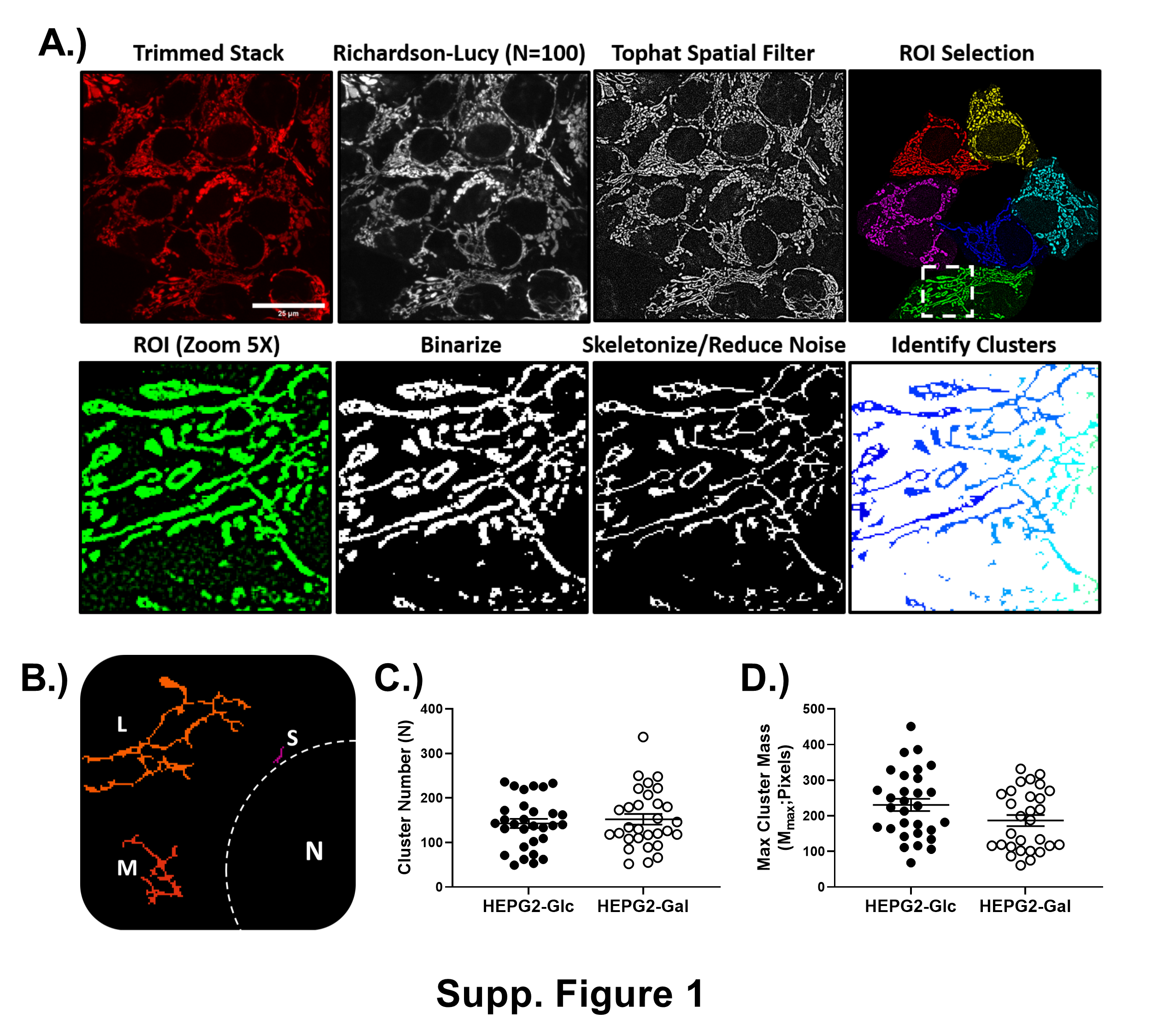

Supplement: Supplementary file 1 — Additional file 1: Supp. Figure 1: Additional intact cell mitochondrial morphology data. (A) Image panel diagram of steps involved in identifying individual mitochondrial cluster distributions in individual cells from laser scanning confocal images of TMRM staining. (B) Image subpanel highlighting the qualitative differences in clusters with either small (S; purple), medium (M; red), or large (L; orange) cluster masses. N represents the nucleus. (C) Mean cluster number (per cell). N = 30 cells/group (D) Mean cluster mass. N = 30 cells/group. Data are mean ± SEM. Means were compared using Student’s t test. Data are mean ± SEM. *p < 0.05. ns = not significant. [file 40170_2021_241_MOESM1_ESM.tif]

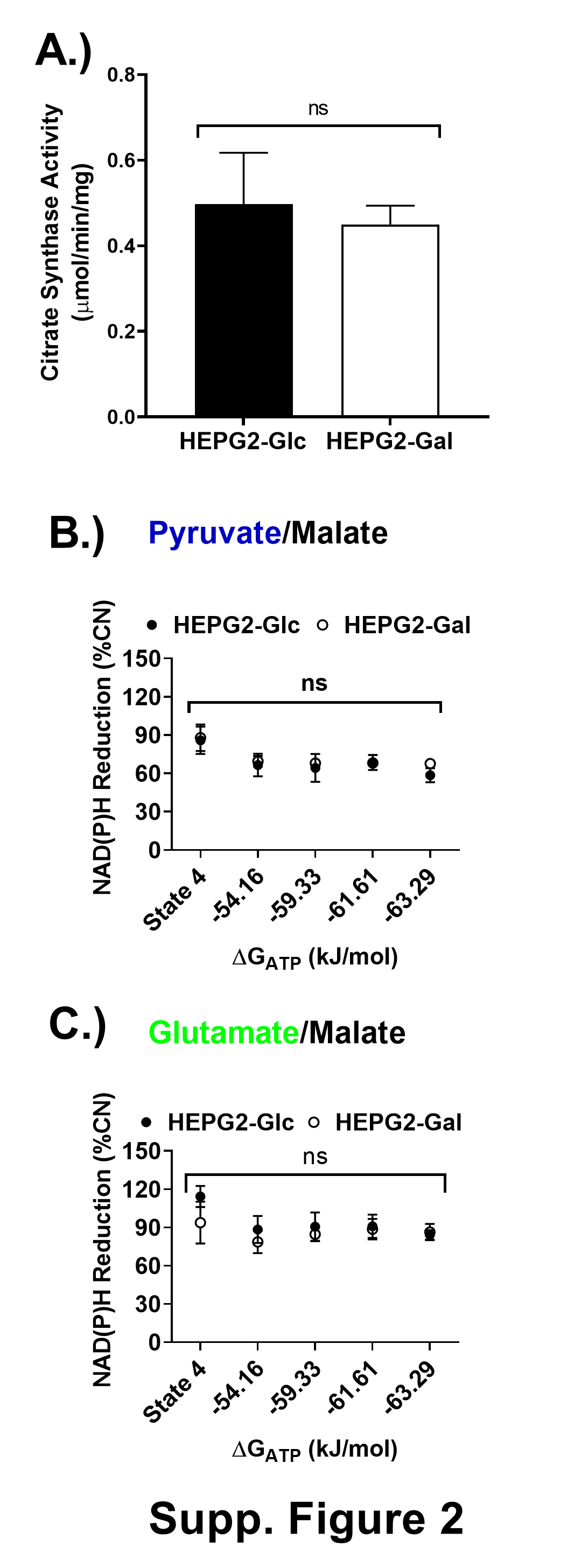

Supplement: Supplementary file 2 — Additional file 2: Supp. Figure 2: Additional isolated mitochondria data. (A) Citrate synthase activity in mitochondria isolated from HEPG2-Glc and HEPG2-Gal cells. (B) NAD(P)+/NAD(P)H autofluorescence is proportional to the redox state of matrix dehydrogenase reactions for pyruvate/malate-supported respiration. (C) NAD(P)+/NAD(P)H autofluorescence is proportional to the redox state of matrix dehydrogenase reactions for glutamate/malate-supported respiration. Data are represented as a percent of the fluorescence measured in the presence of potassium cyanide (10 mM). N = 7/treatment/group. Data are mean ± SEM. Means were compared using Student’s t test (A) and a two-way ANOVA with Sidak’s multiple comparison test (B, C). *p < 0.05. [file 40170_2021_241_MOESM2_ESM.tif]

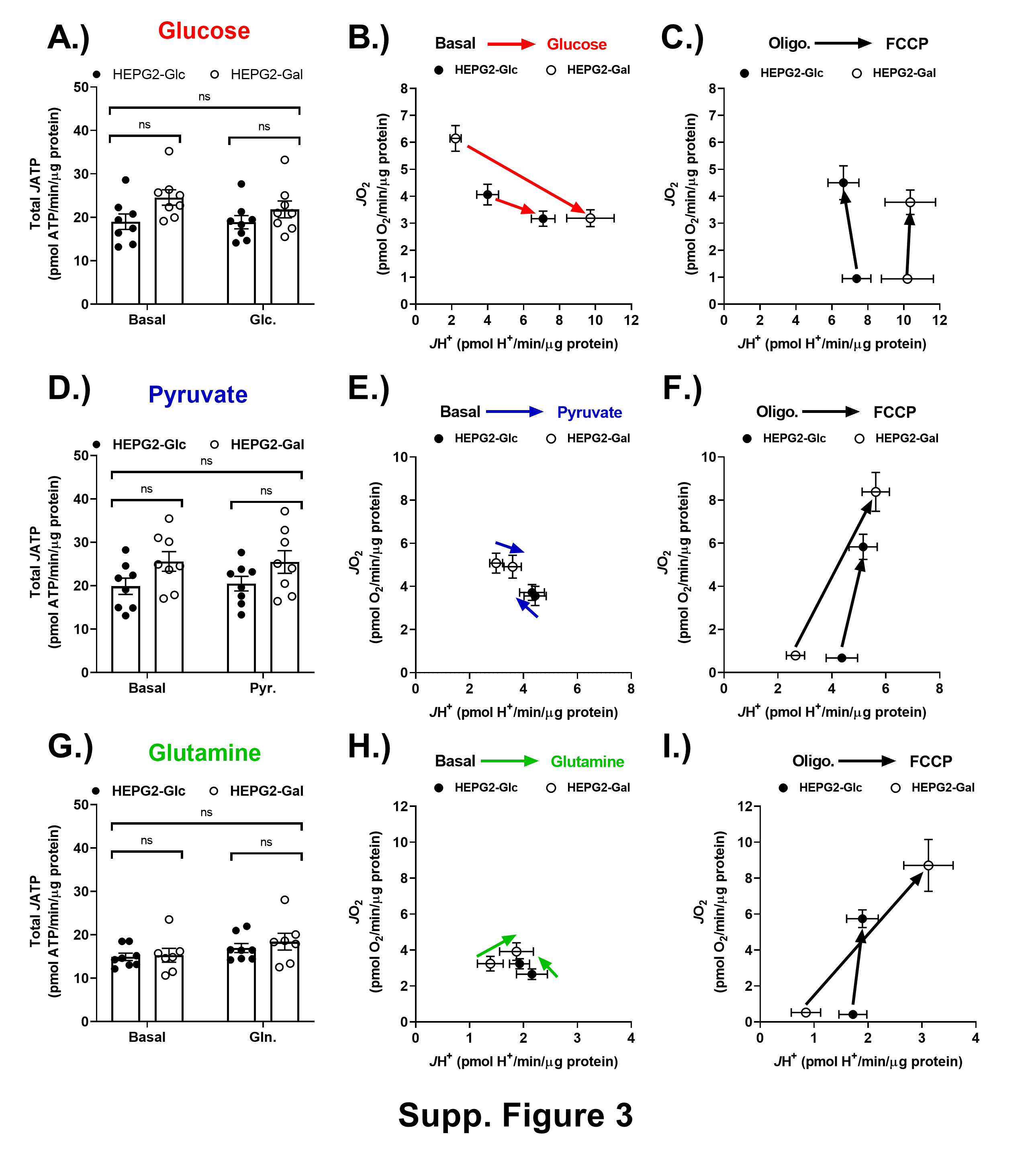

Supplement: Supplementary file 3 — Additional file 3: Supp. Figure 3: Additional intact cell respiration data and total JATP values: (A) Estimated total ATP production rates (JATP) attributable to both OxPhos and aerobic fermentation at baseline and following glucose refeeding. (B) Bivariate plot of respiration rate (JO2) vs. proton efflux rate (JH+) at baseline and following glucose refeeding. (C) Bivariate plot of glucose-supported JO2 vs. JH+ during a high glycolytic flux condition (5 μM Oligomycin) and a high respiratory flux condition (5 μM Oligomycin + 1 μM FCCP). (D) Estimated total ATP production rates (JATP) attributable to both OxPhos and aerobic fermentation at baseline and following pyruvate refeeding. (E) Respiration rate (JO2) vs. proton efflux rate (JH+) at baseline and following pyruvate refeeding. (F) Pyruvate-supported JO2 vs. JH+ during a high glycolytic flux condition and a high respiratory flux condition. (G) Estimated total ATP production rates (JATP) attributable to both OxPhos and aerobic fermentation at baseline and following pyruvate refeeding. (H) Respiration rate (JO2) vs. proton efflux rate (JH+) at baseline and following pyruvate refeeding. (I) Pyruvate-supported JO2 vs. JH+ during a high glycolytic flux condition and a high respiratory flux condition. Data are mean ± SEM. Means were compared using a two-way ANOVA (A, D, G). N = 8/treatment/group. *p < 0.05. ns = not significant. [file 40170_2021_241_MOESM3_ESM.tif]

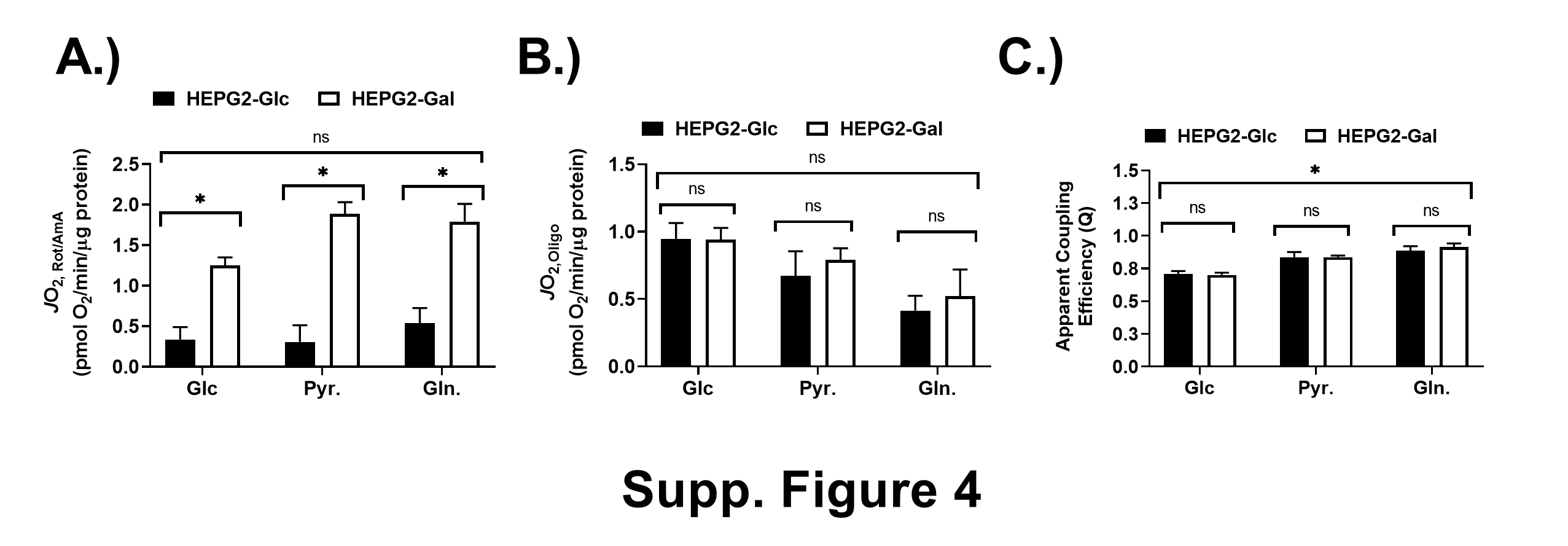

Supplement: Supplementary file 4 — Additional file 4: Supp. Figure 4: Additional intact cell inhibitor data and apparent coupling efficiencies: (A) JO2 following exposure to the NADH oxidoreductase inhibitor rotenone and cytochrome bc1 complex inhibitor antimycin A. (B) JO2 following exposure to FoF1 ATPase inhibitor oligomycin. (C) Apparent coupling efficiencies (Q) for each substrate determined from the fractional change in respiration that occurred following inhibition by oligomycin. Data are mean ± SEM. N = 8/treatment/group. Means were compared using a two-way ANOVA with Sidak’s multiple comparison test. *p < 0.05. ns = not significant. [file 40170_2021_241_MOESM4_ESM.tif]

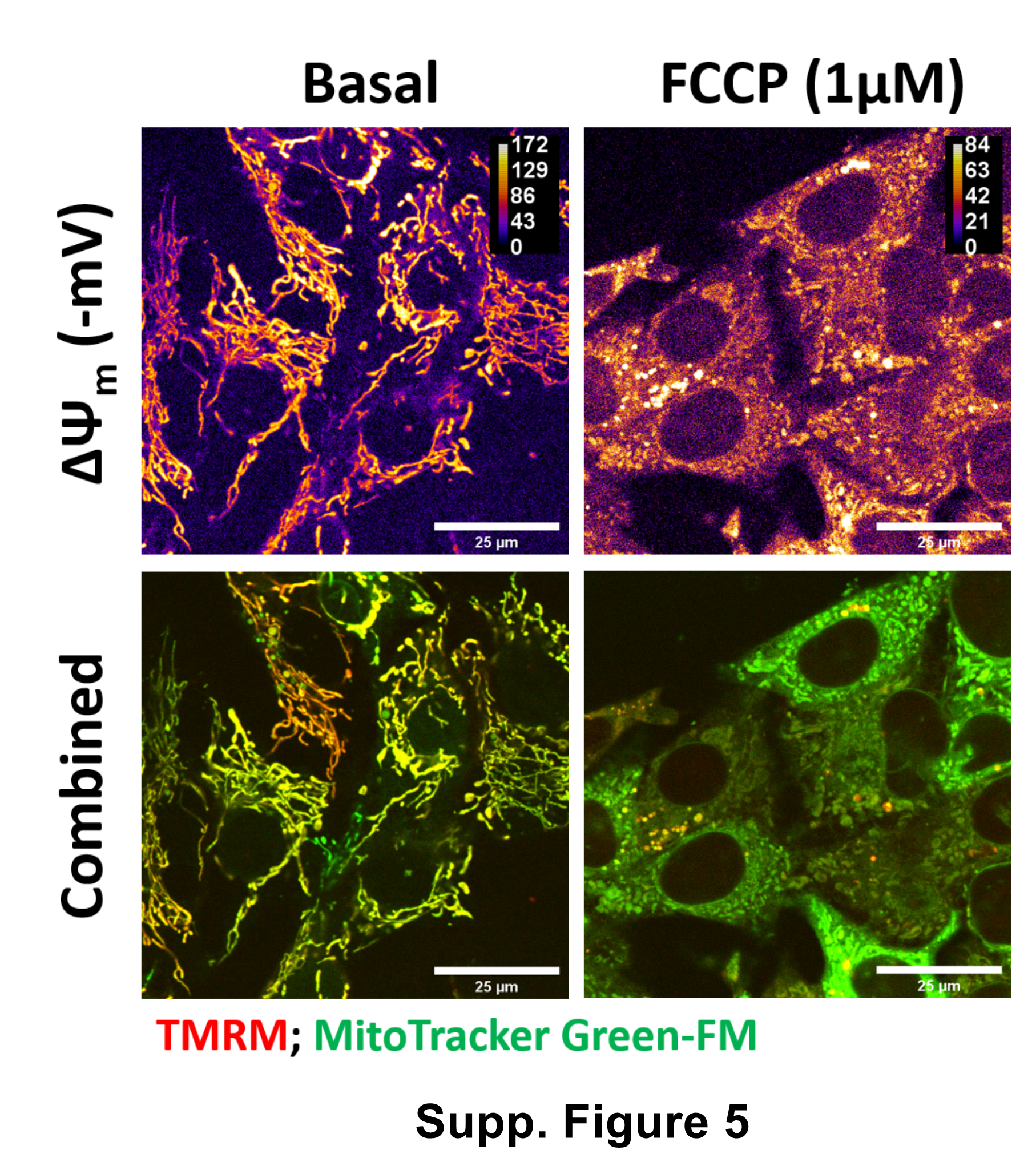

Supplement: Supplementary file 5 — Additional file 5: Supp. Figure 5: Additional intact cell ΔΨm data: Image panel demonstrating the expected redistribution of mitochondrial localized dyes and reduction of ΔΨm in the presence of the cell permeable protonophore (Trifluoromethoxy carbonylcyanide phenylhydrazone; FCCP). Scale bars are 25 μm). [file 40170_2021_241_MOESM5_ESM.tif]
